# The role of anthrax toxin protein receptor 1 as a new mechanosensor molecule and its mechanotransduction in BMSCs under hydrostatic pressure

**DOI:** 10.1038/s41598-019-49100-5

**Published:** 2019-09-02

**Authors:** Baixiang Cheng, Yanzheng Liu, Ying Zhao, Qiang Li, Yanli Liu, Junjun Wang, Yongjin Chen, Min Zhang

**Affiliations:** State Key Laboratory of Military Stomatology & National Clinical Research Center for Oral Diseases & Shaanxi International Joint Research Center for Oral Diseases, Department of General Dentistry and Emergency, School of Stomatology, Fourth Military Medical University, No.145 West Changle Road, Xi’an, 710032 China

**Keywords:** Regeneration, Biomedical engineering

## Abstract

Anthrax toxin protein receptor (ANTXR) 1 has many similarities to integrin and is regarded in some respects as a single-stranded integrin protein. However, it is not clear whether ANTXR1 responds to mechanical signals secondary to the activation of integrins or whether it is a completely new, independent and previously undiscovered mechanosensor that responds to an undefined subset of mechanical signaling molecules. Our study demonstrates that ANTXR1 is a novel mechanosensor on the cell membrane, acting independently from the classical mechanoreceptor molecule integrinβ1. We show that bone marrow stromal cells (BMSCs) respond to the hydrostatic pressure towards chondrogenic differentiation partly through the glycogen synthase kinase (GSK) 3β/β-Catenin signaling pathway, which can be partly regulated by ANTXR1 and might be related to the direct binding between ANTXR1 and low-density lipoprotein receptor-related protein (LRP) 5/6. In addition, ANTXR1 specifically activates Smad2 and upregulates Smad4 expression to facilitate the transport of activated Smad2 to the nucleus to regulate chondrogenesis, which might be related to the direct binding between ANTXR1 and Actin/Fascin1. We also demonstrate that ANTXR1 binds to some extent with integrinβ1, but this interaction does not affect the expression and function of either protein under pressure. Thus, we conclude that ANTXR1 plays a crucial role in BMSC mechanotransduction and controls specific signaling pathways that are distinct from those of integrin to influence the chondrogenic responses of BMSCs under hydrostatic pressure.

## Introduction

Due to its limited blood supply and slow cellular metabolism, articular cartilage is difficult to repair following injury. Identifying methods for repairing defects in cartilage is an ongoing clinical problem^1–3^. The biomechanical environment of articular cartilage can exert a devastating toll on neocartilage that lacks adequate biomechanical properties^4–7^. Because stem cells have higher mechanical sensitivity than adult cells^8^, biomechanical signals may play key roles in regulating the phenotypic differentiation of stem cells^9^. Stem cells can sense mechanical properties and perceive mechanical information that directs broad aspects of cell functions, including lineage commitment. Our previous work showed that stimulation with hydrostatic pressure could effectively activate the chondrogenic potential of BMSCs^10–13^, suggesting that stem cells pretreated with suitable mechanical stimulation could be transformed into “super cells” with high chondrogenic potential that could better survive the growth and secretion rhythm of residual stem cells surrounding a defect after implantation^14^. However, although mechanical stimulation may be able to enhance the regeneration process of articular cartilage^15^, the underlying molecular regulation mechanism remains far from clear.

Research on the mechanisms responsible for the conversion of extracellular mechanical stimuli into biochemical signals has identified a number of possible cell membrane mechanoreceptors, including integrins^16^, G-protein coupled receptors^17^, stretch-activated ion channels^18^, and nonmotile primary cilia^19^. The most classical molecules involved in mechanotransduction are the integrins^20^. When integrins are activated, their ectodomains become extended and upright^21^, and the hybrid domain swings open away from the α-subunits^22,23^. This activation then leads to focal adhesion kinase (FAK) activation, cytoskeletal rearrangement, microfilament protein conformational changes, and finally cell function changes. However, integrin activation alone is necessary but not sufficient for many vital cellular functions, such as cell spreading, cell growth, and proliferation. In recent years, several GPCRs have been shown to respond to different mechanical stimuli *in vitro*^17^ to mediate fluid shear stress-induced endothelial responses, including [Ca^2+^]i transients, the activation of endothelial NO synthase (eNOS), the phosphorylation of PECAM-1 and VEGFR-2, and the activation of SRC and AKTs^24^. Mesenchymal stem cells (MSCs) are a promising cell source for tissue engineering and regenerative medicine strategies. Although our previous work showed that stimulation with hydrostatic pressure could effectively activate the chondrogenic potential of BMSCs, how they initiate the mechanical signaling transduction process among so many mechanotransduction molecules remains unclear. To better understand the mechanical and biological responses and the signal transduction mechanisms of BMSCs in response to stress, stable isotope labeling by amino acid (SILAC) detection in BMSCs was used to screen for differentially expressed signal molecules after mechanical stimulation. We found that the classical membrane mechanoreceptor integrin β1 increased in BMSCs stimulated by hydrostatic pressure. Moreover, the examination of several signal transduction molecules known to be involved in functions related to membrane mechanic sensitivity, such as cell leading, lamellipodium and regulating the actin cytoskeleton, revealed the most significant changes in the expression levels of anthrax toxin protein receptor 1 (ANTXR1), also known as tumor endothelial marker 8 (TEM8) (see Supplementary Materials, Fig. S1 and Tabs S2, S3 and S4), which could be a newly recognized molecule in the field of mechanotransduction.

ANTXR1 was previously discovered as a tumor endothelial marker (TEM), as it is expressed at extremely low levels in normal tissues and is significantly upregulated in tumor tissues^25^. However, the physiological function of ANTXR1 remains unclear. Previous studies have found that ANTXR1 shares many similarities with integrin proteins and is even considered to some extent to be a single-stranded integrin protein. The primary difference between them is that integrins always maintain the alpha beta dimer form, while ANTXR1 can adopt either an open or closed conformation of its von Willebrand A (vWA) domain binding ligand. ANTXR1 can form a complex with the Wnt coreceptor and β-Catenin upstream molecule low-density lipoprotein receptor-related protein (LPR) 6 and therefore has the potential to regulate the Wnt/β-Catenin pathway. In addition, the cytoplasmic tail of ANTXR1 can be directly anchored by the actin cytoskeleton and therefore has a cytoskeletal regulatory effect, which is similar to the mechanical signal transduction pathway of integrins^26–28^. Studies have reported that integrin β1 is a protein with a vWA structure on the cell surface that mediates the death of murine macrophages caused by the anthrax toxin, suggesting that integrin β1, like ANTXR1, could be an anthrax toxin receptor^22,29^. The integrin α4β1 and α5β1 complexes even have anthrax toxin antigen binding capacities very similar to that of ANTXR1. However, whether ANTXR1 is a mechanical signaling sensory molecule like integrin β1 has not yet been determined. It also remains unknown whether the response of ANTXR1 to mechanical stimuli is completely independent of integrins or is secondary to integrin activation.

Here, we show that ANTXR1 is a novel mechanosensor molecule on the cell membrane, and its mechanical sensitivity and mechanotransduction pathways are independent of the classical mechanoreceptor integrinβ1. We also provide evidence that ANTXR1 can directly bind to the coreceptor of the Wnt proteins LRP5 and LRP6 and to the cytoskeleton molecule Actin and its binding protein Fascin1. Furthermore, it partly modulates the mechanobiological upregulation of LRP5, LRP6, phosphorylated glycogen synthase kinase3β (GSK3β), active β-catenin and Smad4 and completely controls the mechanobiological phosphorylation of Smad2, which might, in turn, mediate the mechanical promotion of BMSC chondrogenesis by upregulating the sex determining region Y-box 9 (Sox-9), aggrecan and type II collagen (Col-II) genes. Together, these data clearly demonstrated a previously undiscovered role for ANTXR1 in mechanotransduction.

## Results

### Construction of rat BMSC sheets

**Figure 1:**
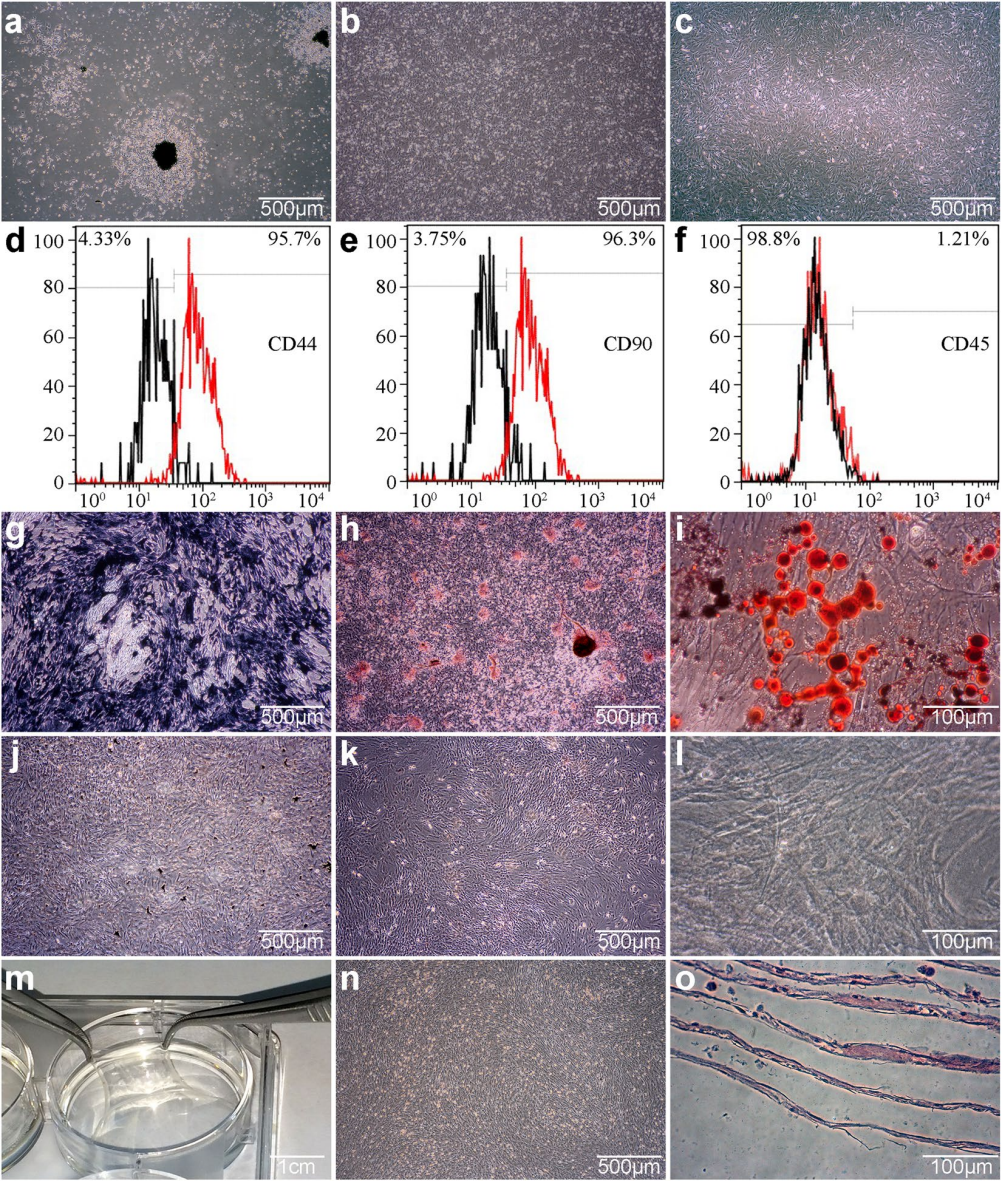
Rat BMSC cultures, identification and cell sheets fabrication. (**a**) Cell morphology after 1 day at passage 0. Scale bar = 500 µm. (**b**) Cell morphology after 7 days at passage 0. Scale bar = 500 µm. (**c**) Cell morphology at passage 3. Scale bar = 500 µm. (**d**–**f**) Assay of cell surface antigens in BMSCs. (**g**) Osteogenic induction of cells after 21 days, ALP staining. Scale bar = 500 µm. (**h**) Osteogenic induction of cells after 21 days, Alizarin Red S staining. Scale bar = 500 µm. (**i**) Adipogenic induction of cells after 14 day, Oil Red O staining. Scale bar = 100 µm. (**j**) Uninduced cells after 21 day, ALP staining. Scale bar = 500 µm. (**k**) Uninduced cells after 21 days, Alizarin Red S staining. Scale bar = 500 µm. (**l**) Uninduced cells after 14 days, Oil Red O staining. Scale bar = 100 µm. (**m**) Macroscopic images of BMSC sheets. Scale bar = 1 cm. (**n**) Cell sheets morphology after 14 days of induction. Scale bar = 500 µm. (**o**) H&E staining of BMSC sheets. Scale bar = 100 µm.

We found that the cells were colony-like after the primary BMSCs from SD rats were cultured for 1 day (Fig. 1a). When the primary cells were cultured until day 7, the cells in the colonies gradually merged and grew over the bottom of the flask (Fig. 1b). After passaging to the third generation, the cells adhered rapidly and showed a uniform, long fusiform shape, and the growth rate tended to be stable (Fig. 1c). Using flow cytometry to assess the BMSC surface markers resulted in a CD44 positive rate 95.7%, a CD90 positive rate of 96.3%, and a CD45 positive rate of 1.21% (Fig. 1d–f). Rat P3 BMSCs were induced with osteogenic induction medium for 21 days and then observed using ALP staining. The cells appeared purple-black, with a linear arrangement (Fig. 1g), a large number of mineralized nodules were formed by Alizarin Red S staining (Fig. 1h). After control cells were cultured for 21 days, ALP staining did not show any positive changes (Fig. 1j). The control cells stained with Alizarin Red S also appeared negative (Fig. 1k). Rat P3 BMSCs were then induced with adipogenic induction medium for 14 days. Oil Red O staining showed a large number of lipid droplets in the cells (Fig. 1i). No lipid droplets were observed in control cells (Fig. 1l). The above results help to determine that the cells we cultured were MSCs. Subsequently, the cells were cultured with membrane-inducing solution for 14 days. Many milky white, membraneous substances formed in the 6-well plates. The cell membrane could be clamped with tweezers, indicating that it has good flexibility (Fig. 1m). Under the microscope, the membrane was composed of densely grown cells and abundant extracellular matrix (Fig. 1n). Hematoxylin and eosin (H&E) staining showed that the cells were multi-layered (Fig. 1o). Cell sheets from the third generation of BMSCs were used for the follow up experiments.

### Mechanosensitivity of ANTXR1 and chondrogenic differentiation of BMSCs

**Figure 2:**
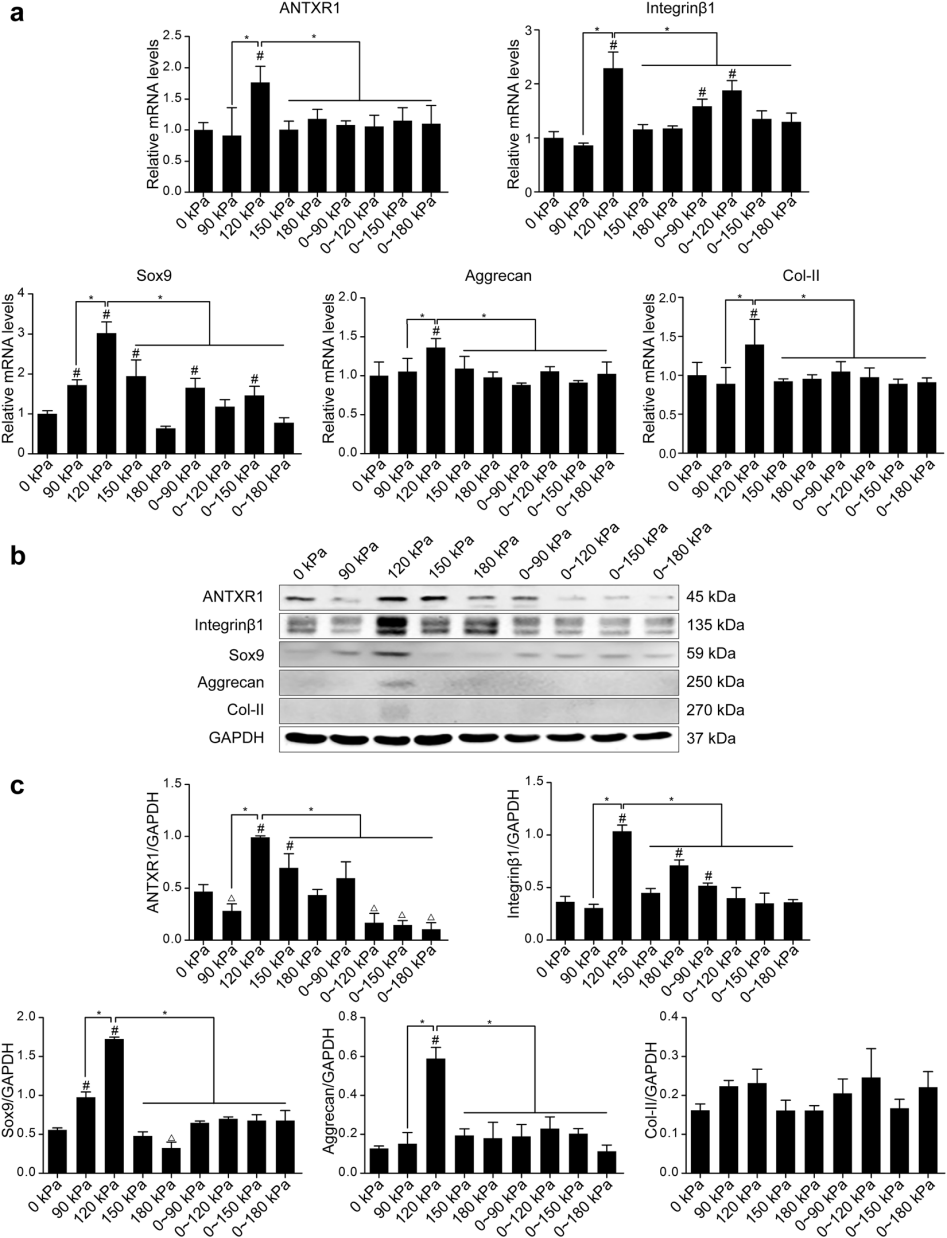
Effects of different pressure stimulating conditions on the expression of ANTXR1 and the chondrogenic potential of BMSC sheets. (**a**) RT-PCR was performed to determine the mRNA expression levels of ANTXR1, integrinβ1, and 3 chondrogenic markers in BMSC sheets treated with 8 different hydrostatic pressure conditions for 1 h. (**b**) BMSC sheets were stimulated with 8 different hydrostatic pressure conditions for 1 h. Extracts from the cytoplasmic fractions were analyzed by western blotting analysis to determine the protein expression levels of ANTXR1, integrinβ1, and 3 chondrogenic markers, Sox-9, aggrecan, and Col-II. Expression levels relative to that for GAPDH were derived using Quantity One density analysis. BMSCs treated with no pressure was used as a control. (**c**) Quantitative analysis of the western blotting bands by ImageJ software. Four independent assays were performed for each group. Data presented as the mean ± SD. ^#^*P* < 0.05 represents a significant increase compared with the control group. ^*^P < 0.05 represents a significant difference compared with the indicated groups. ^△^*P* < 0.05 represents a significant decrease compared with the control group.

Six-well plates containing BMSC sheets were randomly divided into two groups according to the type of pressure applied: one group under static pressure and one under dynamic pressure stimulation. The static pressure group was randomly subdivided into four subgroups that received 90 kPa, 120 kPa, 150 kPa and 180 kPa, and the dynamic pressure group was randomly subdivided into four subgroups that received 0–90 kPa, 0–120 kPa, 0–150 kPa, and 0–180 kPa, resulting in a total of 8 subgroups, with the 0 kPa group as the control. The pressure time for all the groups was set at 1 h. RT-PCR results showed that among the 8 pressure loading groups, the gene expression level of ANTXR1 in BMSCs significantly increased under 120 kPa of static pressure (*P* < 0.05). However, no significant changes in ANTXR1 gene levels were observed for the other pressure loading groups (*P* > 0.05). In contrast, the integrin β1 gene significantly increased in three groups, 120 kPa static pressure and 0–90 kPa and 0–120 kPa dynamic pressure (*P* < 0.05), with the largest increase in the 120 kPa static pressure group (*P* < 0.05 *vs*. 0–90 kPa and 0–120 kPa groups). The chondrogenic genes Aggrecan and Col-II were significantly upregulated only in the 120 kPa static pressure group (*P* < 0.05). The chondrogenesis marker Sox-9 showed significant variations in several pressure groups (*P* < 0.05), with its highest level in the 120 kPa static pressure group (*P* < 0.05) (Fig. 2a). Western blotting analysis showed the same pattern observed for gene detection. The protein expression levels of integrin β1, ANTXR1, Sox-9 and Aggrecan were highest in the 120 kPa static pressure group (*P* < 0.05), while Col-II protein expression did not show any significant variations in any of the experimental groups (*P* > 0.05) (Fig. 2b,c). Based on these results, the pressure loading condition of 120 kPa static pressure for 1 h was selected for subsequent experiments.

### Role of ANTXR1 in the mechanobiological responses of BMSCs

**Figure 3:**
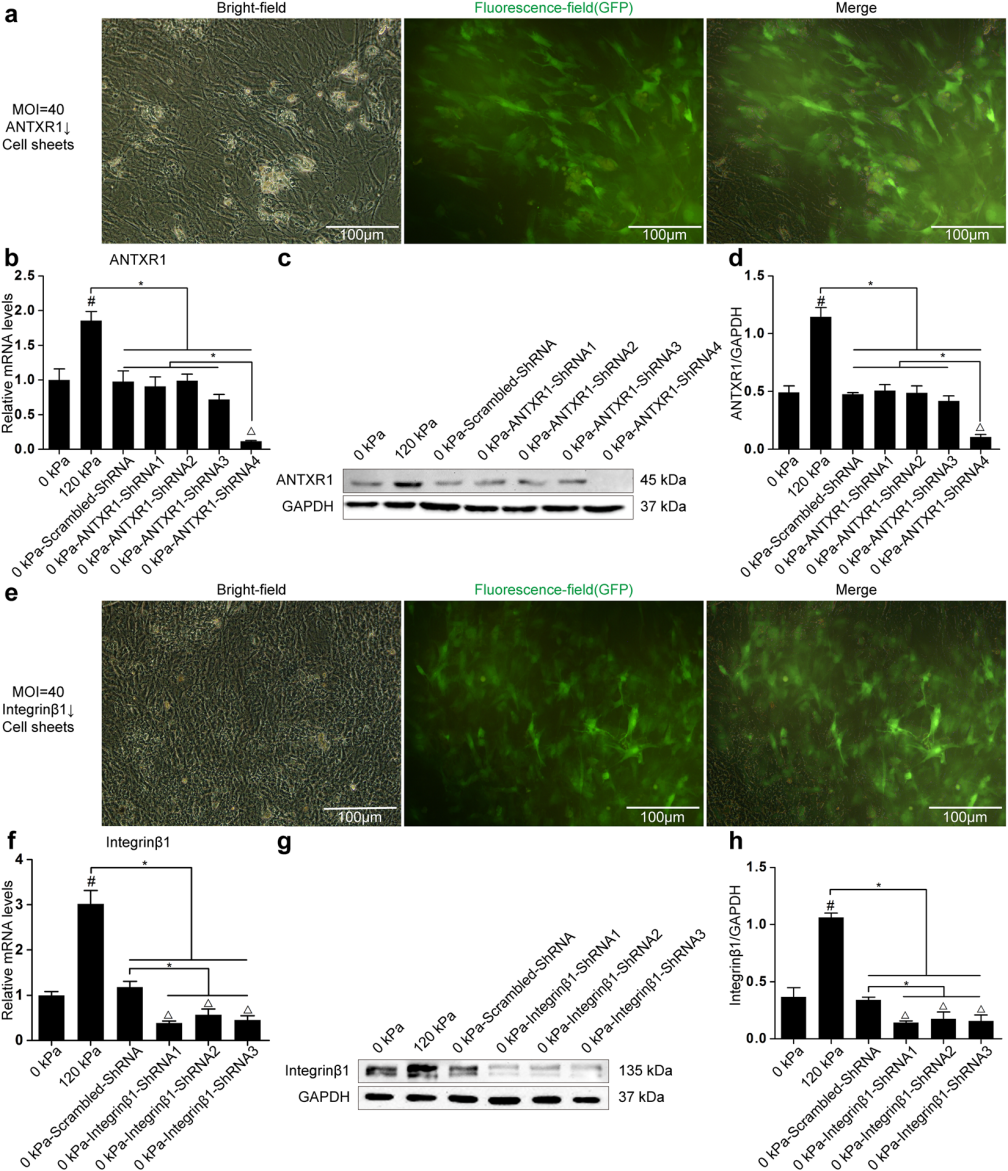
Establishment of ANTXR1-downregulated or integrinβ1-downregulated BMSC sheets. (**a**) Fluorescent microscopy images of BMSC sheets transfected with ANTXR1-shRNA at MOI = 40. Scale bar = 500 µm. (**b**) RT-PCR was performed to determine the mRNA expression levels of ANTXR1. (**c**,**d**) Western blotting was performed to determine the protein expression levels of ANTXR1, followed by the quantitative analysis of the western blotting bands by ImageJ software. (**e**) Fluorescent microscopy images of BMSC sheets transfected with integrinβ1-shRNA at MOI = 40. Scale bar = 500 µm. (**f**) RT-PCR was performed to determine the mRNA expression levels of integrinβ1. (**g**–**h**) Western blotting was performed to determine protein expression levels of integrinβ1, followed by the quantitative analysis of the western blotting bands by ImageJ software.

After inducing the ANTXR1-shRNA lentivirus (MOI = 40) transfected BMSCs to form cell sheets, the transfection efficiency was determined by fluorescence microscopy to be greater than 80% (Fig. 3a). Cell sheets composed of BMSCs transfected with ANTXR1-shRNA were further tested. RT-PCR showed that the expression of the ANTXR1 gene in the cell sheets transfected with the scrambled lentiviral sequence and the 0 kPa-ANTXR1-ShRNA1-3 group did not differ significantly from that in the blank control group (*P* > 0.05), while the expression of the ANTXR1 gene in the 0 kPa-ANTXR1-ShRNA4 group was lower than in the blank control group and the other 4 transfection groups (*P* < 0.05) (Fig. 3b). Western blotting analysis showed that the protein expression level of ANTXR1 in the untransfected cell sheets subjected to 120 kPa for 1 h was higher than those in the other experimental groups (*P* < 0.05). The expression levels of ANTXR1 protein in the cell sheets transfected with the scrambled lentiviral sequence and the 0 kPa-ANTXR1-ShRNA1-3 group were not significantly different from that in the blank control group (*P* > 0.05), while ANTXR1 protein expression in the 0 kPa-ANTXR1-ShRNA4 group was lower than in the blank control group and the other 4 transfection groups (*P* < 0.05) (Fig. 3c,d). The 0 kPa-ANTXR1-ShRNA4 lentivirus condition was selected as the most effective for subsequent transfection experiments.

Similarly, Integrinβ1-shRNA lentivirus (MOI = 40) was transfected into P1 rat BMSCs. After 72 h, the LSCM results showed that the lentiviral transfection efficiency was over 80%. After culturing the transfected cells into cell sheets, the transfection efficiency observed by fluorescence microscopy remained above 80% (Fig. 3e). The integrinβ1-shRNA cell sheets were further investigated. RT-PCR showed that integrinβ1 gene synthesis in the cell sheets transfected with scrambled lentiviral sequence was not significantly changed (*P* > 0.05), while the integrinβ1 gene expression levels in the three 0 kPa-integrinβ1-shRNA groups were lower than that in the blank control group (*P* < 0.05) (Fig. 3f). The results of western blotting analysis showed that the protein expression level of integrinβ1 in the untransfected cell sheets subjected to 120 kPa for 1 h was higher than the levels in other experimental groups (*P* < 0.05). The protein expression level of the cell sheets transfected with the scrambled lentiviral sequence was not significantly different from that of the blank control group (*P* > 0.05), and the integrinβ1 protein expression in the three 0 kPa-integrinβ1-shRNA groups was lower than that in the blank control group (*P* < 0.05) (Fig. 3g,h). Based on these results, the 0 kPa-integrinβ1-shRNA1 lentivirus was selected as the most effective virus for subsequent transfection experiments.

**Figure 4:**
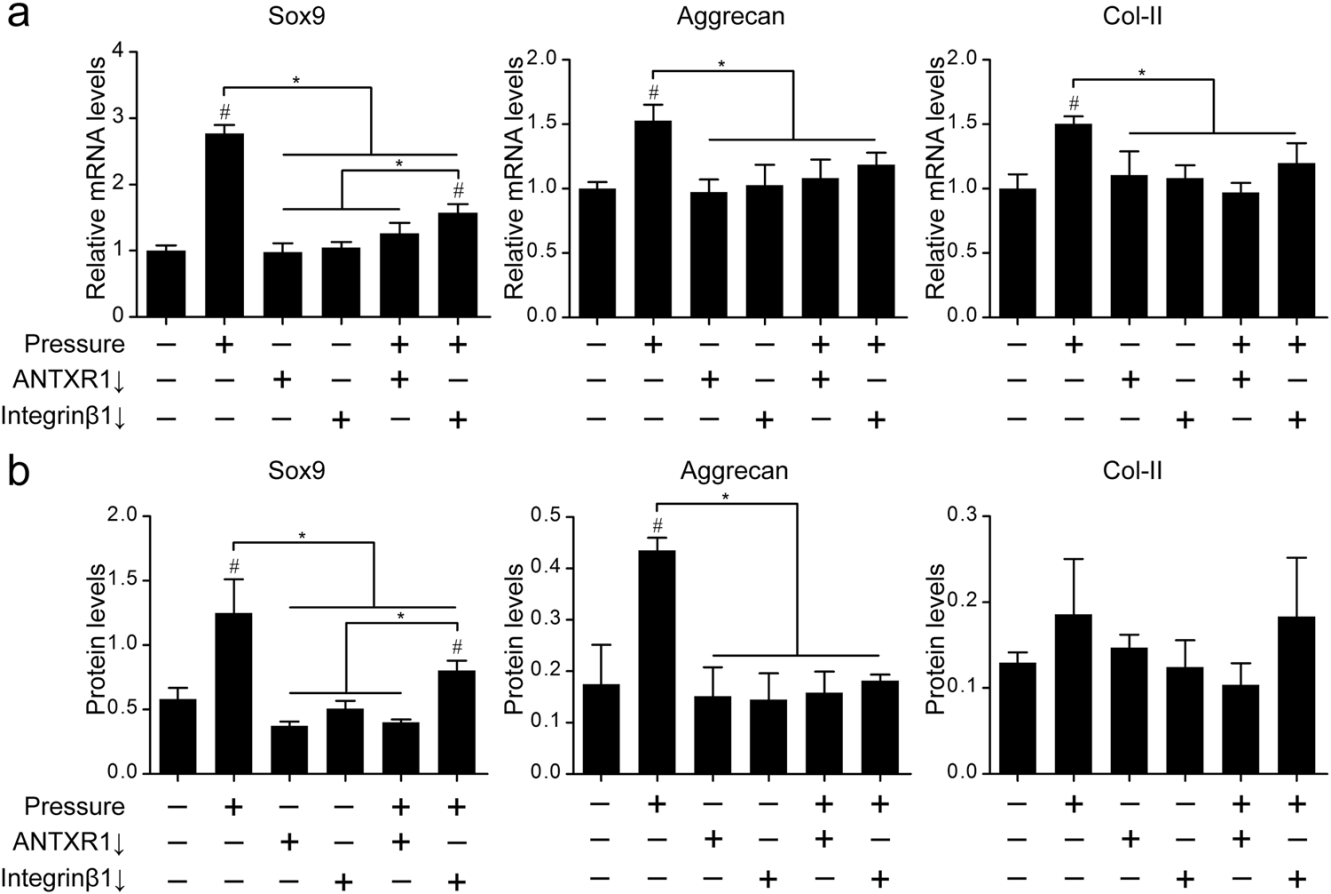
Knockdown of ANTXR1 or integrinβ1 partly inhibits mechiancally promoted chondrogenic differentiation of BMSs. (**a**) RT-PCR was performed to determine the mRNA expression levels of Sox-9, aggrecan and Col-II. (**b**) Western blotting was performed to determine the protein expression levels of Sox-9, aggrecan and Col-II. (**c**) Quantitative analysis of the western blotting bands by ImageJ software. Four independent assays were performed for each group. Data presented as the mean ± SD. ^#^*P* < 0.05 represents a significant increase compared with the control group. ^*^P < 0.05 represents a significant difference compared with the indicated groups. ^△^*P* < 0.05 represents a significant decrease compared with the control group.

Hydrostatic pressure was performed on the lentivirus-transfected BMSC sheets. The results of both RT-PCR and western blotting showed that, the expression levels of Sox-9, aggrecan and Col-II gene (Fig. 4a) and protein (Fig. 4b) in the untransfected rat BMSCs sheets under pressure were significantly increased (*P* < 0.05, *vs*. blank control). After the integrinβ1 in rat BMSCs sheets were stably downregulated and then pressurized, the expression levels of aggrecan and Col-II gene and protein were decreased and back to the level of the control group. The expression of Sox-9 gene and protein was also partially inhibited, which was reflected as a significant decrease compared to the cells in the group without viral transfection, however, it was maintained at a level higher than that of the blank control group (*P* < 0.05, *vs*. blank control). After ANTXR1 was downregulated, the BMSCs sheets were further pressurized, and the expression levels of the gene and protein of the three cartilage markers were no longer elevated, and remained at the level of the control (*P* > 0.05, *vs*. blank control).

### Relationship between ANTXR1 and integrinβ1 during mechanotransduction

**Figure 5:**
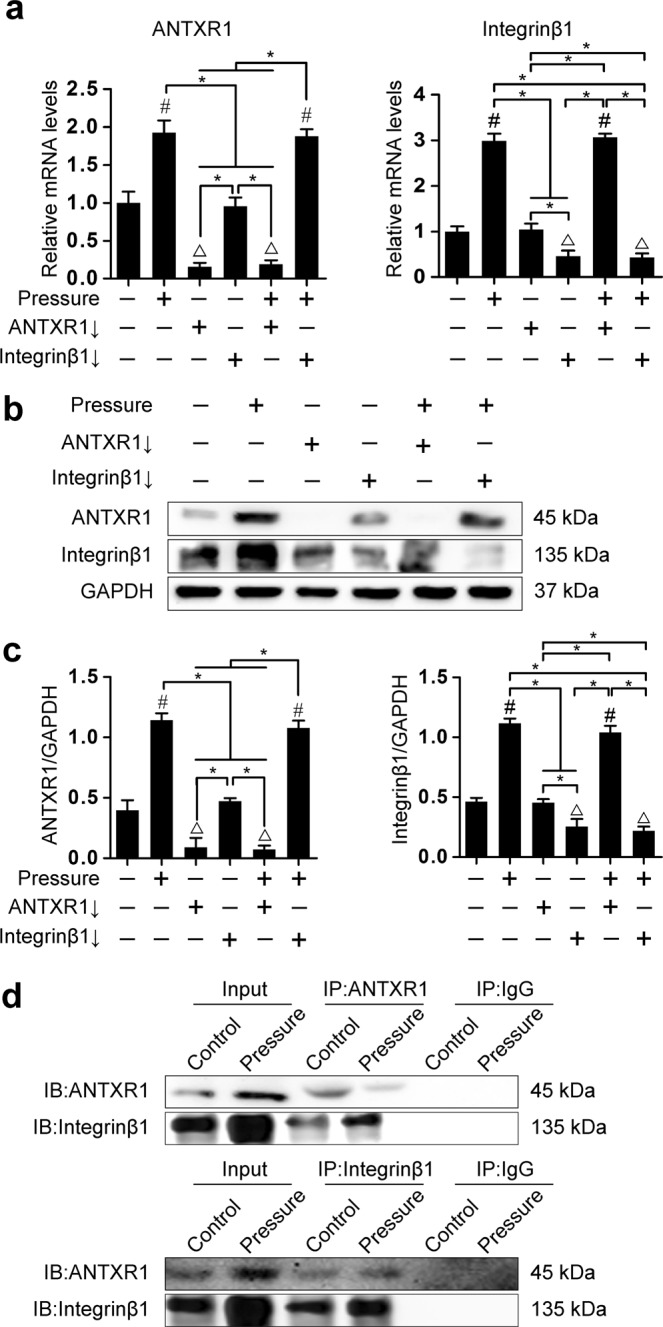
Association between ANTXR1 and integrinβ1. (**a**) RT-PCR was performed to determine the mRNA expression levels of ANTXR1 and integrinβ1. (**b**) Western blotting was performed to determine the protein expression levels of ANTXR1 and integrinβ1. (**c**) Quantitative analysis of the western blotting bands by ImageJ software. (**d**) Interactions between ANTXR1 and integrinβ1 were demonstrated by Co-IP assays. Western blots of the inputs and immunoprecipitates were analyzed using the indicated antibodies. Four independent assays were performed for each group. Data presented as the mean ± SD. ^#^*P* < 0.05 represents a significant increase compared with the control group. ^*^P < 0.05 represents a significant difference compared with the indicated groups. ^△^*P* < 0.05 represents a significant decrease compared with the control group.

The relationship between ANTXR1 and integrinβ1 was further verified using the rat BMSCs sheets successfully transfected with lentivirus. The results of RT-PCR showed that, the BMSCs sheets after the downregulation of ANTXR1 were subjected to pressure-loading, and the expression level of ANTXR1 gene was no longer increased with the pressure stimulation (*P* > 0.05), but the integrinβ1 gene showed a significant upregulation after pressure stimulation regardless of whether ANTXR1 was downregulated or not (*P* < 0.05). Vice versa, when the integrinβ gene was downregulated and the BMSCs sheets were subjected to pressure-loading, the expression of integrinβ1 gene was significantly inhibited (*P* > 0.05), but the ANTXR1 gene was still significantly upregulated under pressure stimulation (*P* < 0.05) (Fig. 5a). The results of western blotting analysis showed that the expression levels of the integrinβ1 protein in BMSC sheets where ANTXR1 was downregulated were significantly different between two group with or without pressure stimulation (*P* < 0.05). Similarly, in the BMSC sheets where integrinβ1 was downregulated, the expression levels of ANTXR1 protein were significantly different between the two groups with or without pressure stimulation (*P* < 0.05) (Fig. 5b,c).

To further determine whether there is a direct relationship between the molecular structures of ANTXR1 and integrinβ1 in BMSCs, a Co-IP assay was performed, and the results showed that pressure stimulation of 120 kPa for 1 h could effectively upregulate the expression levels of both ANTXR1 and integrinβ1. In the Co-IP reaction, using either ANTXR1 or integrinβ1 as the bait protein, the ANTXR1 and integrinβ1 proteins were detected in both the blank cell sheets group and the pressure-loaded cell sheets group. When IgG was used as bait protein, neither the ANTXR1 protein nor the integrinβ1 protein were detected in either the blank cell sheets group or the pressure-loaded cell sheets group. These results indicated that, under culture conditions with or without pressure, ANTXR1 and integrinβ1 experienced binding activity (Fig. 5d).

**Figure 6:**
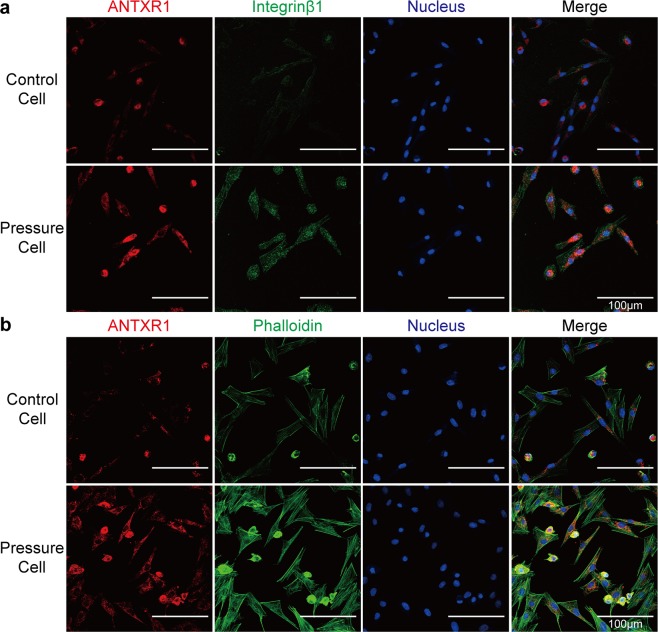
ANTXR1 interacted with integrinβ1 and the cytoskeleton. (**a**) Co-localization between ANTXR1 and integrinβ1 was detected by immunofluorescence in BMSCs from the 0 kPa control group and from the group subjected to 120 kPa of static pressure for 1 h. ANTXR1 was stained red, integrinβ1 was stained green, and nuclei were stained blue. Scale bar = 100 µm. (**b**) Co-localization between ANTXR1 and the F-actin was detected by immunofluorescence in BMSCs from the 0 kPa control group and from the group subjected to 120 kPa of static pressure for 1 h. ANTXR1 was stained red, F-actin was stained green by phalloidin and nuclei were stained blue. Scale bar = 100 µm. All experiments were repeated at least three times.

Immunofluorescence was used to validate where ANTXR1 (red fluorescence) and integrinβ1 (green fluorescence) were localized in BMSCs, with or without hydrostatic pressure stimulation. The results indicated that ANTXR1 partially co-localized with integrinβ1 in BMSCs (Fig. 6a). As a classical signal molecule downstream to integrinβ1, the fluorescence of F-actin was also observed by phalloidin staining (green fluorescence). It indicated that the fluorescence of F-actin was significantly strengthened under mechanical stimulation. The red fluorescence of ANTXR1 and the green fluorescence of phalloidin were also observed to greatly overlap in the cytoplasm of BMSCs, indicating that ANTXR1 co-localized with the F-actin in BMSCs (Fig. 6b). Besides, to well-illustrate the co-localization, the quantitative analysis for the Fig. 6., was presented in the Tables S7 and S8 of Supplementary Materials.

### ANTXR1-related downstream mechanotransduction signaling molecules

**Figure 7:**
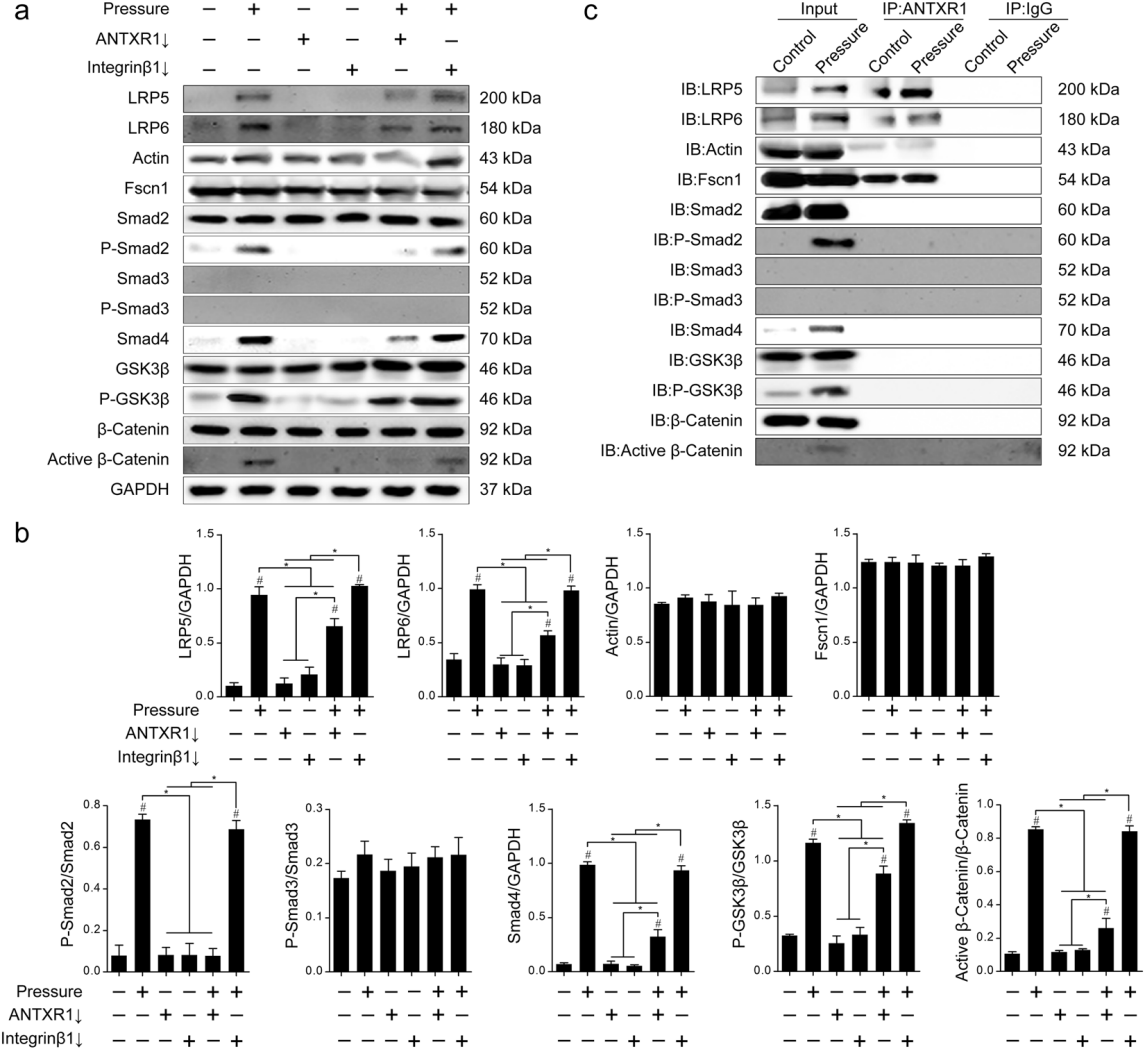
The downstream signaling pathway of ANTXR1. (**a**) Western blotting was performed to determine the protein expression levels of LRP5, LRP6, Actin, Fscn1, Smad2, P-Smad2, Smad3, P-Smad3, Smad4, GSK3β, P-GSK3β, β-Catenin and active β-Catenin. (**b**) Quantitative analysis of the western blotting bands by ImageJ software. (**c**) ANTXR1 interacts with LRP5, LRP6, Actin and Fscn1 in BMSCs, as determined by IP assays. Four independent assays were performed for each group. Data presented as the mean ± SD. ^#^*P* < 0.05 represents a significant increase compared with the control group. ^*^P < 0.05 represents a significant difference compared with the indicated groups.

To further detect the downstream signaling pathway of ANTXR1 the expression and phosphorylation levels of six signaling molecules, including LRP5, LPR6, GSK3β, β-Catenin, Actin and Fscn1, were examined, as these proteins have all been previously reported in the literature as being related to ANTXR1. The activation of the Smad2, Smad3, and Smad4 signaling molecules, which are closely related to chondrogenic differentiation, was also examined. The results of western blotting showed that 120 kPa of pressure for 1 h could induce the protein expression of LRP5, LPR6, Smad4, phosphorylation of Smad2 and GSK3β, ad activation of β-Catenin (*P* < 0.05, *vs*. blank control) in BMSC sheets. The protein expressions of Actin, Fscn1, and phosphorylation of Smad3 did not change significantly before and after pressure stimulation. The downregulation of either ANTXR1 or integrinβ1 alone did not affect the protein expression,phosphorylation or activation of the detected nine signaling molecules, including LRP5, LPR6, Smad2, Smad3, Smad4, GSK3β, β-Catenin, Actin, and Fscn1. After downregulating ANTXR1 in BMSC sheets and then subjecting these sheets to static pressure at 120 kPa for 1 h, the protein expressios of LRP5, LPR6, Smad4, phosphorylation of GSK3β, and activation of β-Catenin decreased significantly compared to those in cell sheets without ANTXR1 downregulation that were subjected to pressure (*P* < 0.05, *vs*. pressure group); however, these protein variations remained significantly increased compared to those in the blank control group without pressure stimulation (*P* < 0.05, *vs*. blank control). The results suggested that the upregulation, phosphorylation or activation of five signaling molecules, LRP5, LPR6, Smad4, GSK3β, and β-Catenin, under pressure stimulation was partially dependent on ANTXR1. In addition, it was noticed that, in BMSC sheets with downregulated ANTXR1 that were subjected to 120 kPa of pressure for 1 h, the phosphorylation of Smad2 was completely decreased to the level observed for the blank control (*P* < 0.05, *vs*. Pressure group; *P* > 0.05, *vs*. blank control). This result suggested that the Smad2 acvtivation under the pressure was completely dependent on ANTXR1. To compare the similarities and differences between the downstream signaling pathways of ANTXR1 with those of integrinβ1, the expression or activation of these signaling molecules was examined after the downregulation of integrinβ1 in BMSCs sheets subjected to 120 kPa of static pressure for 1 h. In contrast with the downregulation of ANTXR1, the downregulation of integrinβ1 did not significantly change the expression of LRP5, LPR6, P-Smad2, Smad4, P-GSK3β and active β-Catenin following 120 kPa of pressure for 1 h when compared with the simple pressure group (*P* > 0.05, *vs*. pressure group), suggesting that the variation of these six signaling molecules by pressure stimulation is regulated by ANTXR1 but not by integrinβ1 (Fig. 7a,b).

To further explore whether ANTXR1 has direct binding relationships with any of its possible downstream signaling molecules, LRP5, LPR6, GSK3β, β-Catenin, Actin and Fscn1, or with three chondrogenesis-related signaling molecules, Smad2, Smad3, and Smad4, Co-IP experiments were performed. The results showed that, when ANTXR1 was used as the bait protein, LRP5, LRP6, Actin and Fscn1 proteins were detected in both the blank cell sheets group and the pressure-loaded cell sheets group, while the other two ANTXR1-related signaling molecules, GSK3β and β-Catenin, and the three chondrogenesis-related signaling molecules, Smad2, Smad3, and Smad4, had no direct binding relationship with ANTXR1. When IgG was used as the bait protein, none of these proteins were detected in either the blank cell sheets group or the pressure-loaded cell sheets group (Fig. 7c).

## Discussion

Cell mechanics research can mostly be divided into three categories depending on the nature of the force applied: pressure, tension, and shear force. The type of force is selected according to the different stress modes of tissue cells *in vivo*. It has been noted that true compressive loading of MSCs seems to be beneficial for the production of a nonfibrous, cartilage-like matrix, in contrast to tensile loading^30^. Compressive loading is therefore adopted for most biomechanical environment simulation for *in vitro* cartilage research and can be further divided into dynamic and static pressure loading. However, the details are specific to each cartilage-related cell mechanics study; there are no uniform standards for *in vitro* biomechanical conditions due to the different biomechanical devices, different target tissues, and different subsequent research purposes. It has been reported that compressive stress of 7.58 MPa for 4 h every day could induce partial differentiation of bone marrow mesenchymal stem cells into chondrocytes after 2 weeks of continuous loading^31^. Miyanishi *et al*. found that 0.1 MPa compressive stress can increase the expression of the Sox9 gene, while 10 MPa compressive stress can significantly upregulate the expression of Col2α1 protein^32^. Li *et al*. reported that dynamic fluid pressure (13–36 kPa, 0.25 Hz) significantly increased the gene expression of Col2α1 and aggrecan^33,34^. In addition, mesenchymal stem cell growth and cartilage differentiation could also be affected by the osmotic pressure of the medium^35^. Our series of studies is dedicated to studying the regeneration and repair of the stress-sensitive cartilage of the temporomandibular joint by BMSCs. First, the biomechanical characteristics of TMJ articular cartilage were explored by finite element analysis, which showed that the stress in the condylar cartilage was compressive under normal occlusion and equal to approximately 300 kPa^36^. Therefore, in our previous studies on the pressure-induced mechanobiology of primary chondrocytes using a hydraulic pressure-controlling cellular strain unit, we limited the range of pressure on the cells from 30 to 300 kPa^10–14^. In the present study, we detected the mechanotransduction of BMSCs in cell sheets, which will be used as a transplant for further tissue-engineering cartilage regeneration, rather than the BMSCs in monolayers that we studied previously. Therefore, the pressure conditions used in the present study were rescreened, and the appropriate pressure condition was established to be 120 kPa for 1 h.

**Figure 8:**
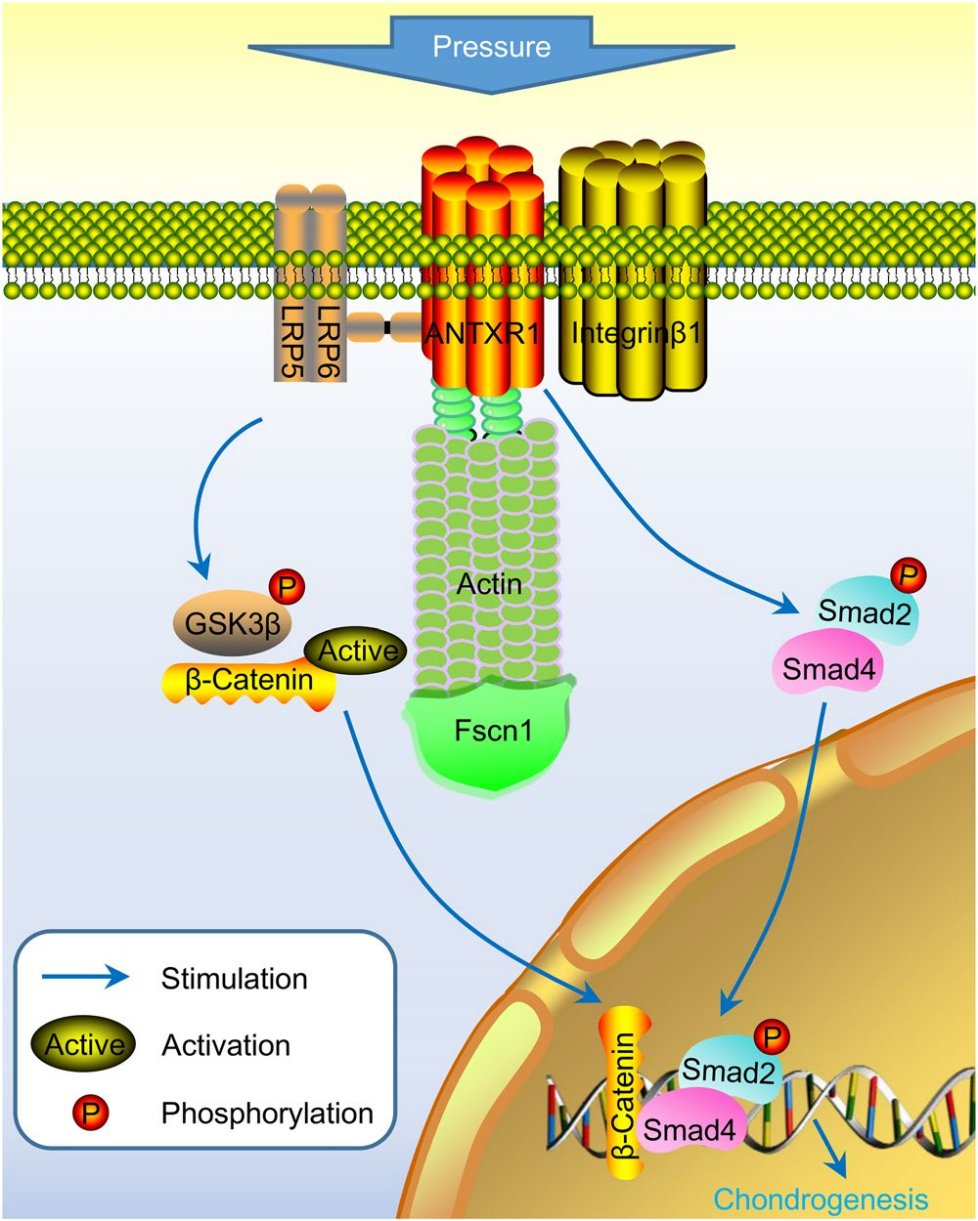
Schematic drawing summarizing the possible role of ANTXR1 on chondrogenic effects in BMSC sheets under hydrostatic pressure.

When mechanical stimulation is sensed by stem cells, a series of signal pathways related to mechanical signal transduction are activated, and stem cell proliferation, migration and differentiation are modulated. Mechanical stimulation can be detected by multiple mechanoreceptors, including SAC^37^, annexin V^38,39^, CD44^40^ and integrins^20,41^. To the best of our knowledge, this study provides the first physiological evidence that ANTXR1 is a novel mechanosensor molecule on the cell membrane that is completely independent of the classical mechanoreceptor molecule integrinβ1. Traditionally, to categorize a protein as a mechanosensor, the following criteria should be met^42^: The protein must be expressed in the correct cells; it must be essential for the immediate signaling response of cells to the relevant force; and it must be activated by the relevant mechanical force when expressed in heterologous cells or in reconstituted lipid bilayers^43^. Here, we revealed that ANTXR1 is expressed in BMSCs that exhibit a significant response to hydrostatic pressure by undergoing chondrogenic differentiation and is required for the hydrostatic pressure sensitivity of BMSCs. We also provide evidence that ANTXR1 can directly bind to the coreceptor of the Wnt proteins LRP5 and LRP6, and to the cytoskeleton molecule Actin and its binding protein Fascin1. Furthermore, it partly modulates the mechanobiological upregulation of LRP5, LRP6, p-GSK3β, active β-catenin and Smad4 and completely controls the mechanobiological phosphorylation of Smad2, which in turn promotes the chondrogenesis of BMSCs by upregulating the Sox-9, aggrecan and Col-II genes. Finally, we demonstrated that the mechanical sensitivity and mechanotransduction pathways of ANTXR1 are independent of the classical mechanoreceptor molecule integrinβ1 (Fig. 8).

To date, most research regarding ANTXR1/TEM8 has been limited to examining the toxicological effects of the anthrax toxin and the roles of ANTXR1/TEM8 in stem cell adhesion and tumor cell proliferation, while the role of ANTXR1/TEM8 as a mechanical signal sensor has not been addressed in the current literature. The downstream mechanical signal transduction pathway initiated after ANTXR1 is subjected to mechanical stimulation and the subsequent conversion of the mechanical signal into a biological signal remain entirely unknown. Some scholars have found that the LRP6, Wnt, and TGF-β pathways are associated with the function of ANTXR1^26,27,44,45^ and that ANTXR1 can form a complex with LRP6, which can interact with and activate the Wnt/β-Catenin signaling pathway^26,27^. In the preliminary study conducted by our team, the classical Wnt/β-Catenin signaling pathway, which plays an important role in the promotion of chondrogenesis, was found to be effectively activated in BMSCs subjected to pressure (Fig. S7). Although this study is not the first to reveal the relationship between ANTXR1 and LRP6 molecules, it is the first to report that ANTXR1 has an important regulatory effect on the mechanobiological response of LRP5 and that LRP5 and LRP6 are both involved in the mechanotransduction initiated by ANTXR1, which in turn activates GSK3β and β-catenin to transfer the mechanical signal into the nucleus. Therefore, we speculated that the mechanobiological effects induced by ANTXR1 are exerted partly through the classical pathway of Wnt/β-catenin signaling. However, we also note that the activation of classical Wnt/β-catenin signaling under mechanical pressure is modulated only partly by ANTXR1. Other upstream molecules also modulate the mechanical response of β-catenin signaling.

ANTXR1 has been reported to be a transmembrane protein with an intracellular actin cytoskeleton binding site and to plays a regulatory role in stem cell migration^46–48^. Go *et al*. proposed that cytoskeletal dynamics regulate the functions of ANTXR1 and its association with the extracellular matrix^47^. Fascin actin-bundling proteins (Fscns) cross-link filamentous actin into tightly packed parallel bundles and play a central role in the architectural maintenance and functions of cell protrusions^49,50^. The preliminary study conducted by our team found that under pressure stimulation, the expression of F-actin was upregulated, the stress fibers assembled, and JNK was phosphorylated^51^. In addition, this effect was regulated by Rac1, a member of the cytoskeletal regulatory protein family^13^. The phalloidin staining in the present work further suggested a clear colocalization relationship between ANTXR1 and F-actin, and Co-IP experiments also confirmed that ANTXR1 interacts with Actin and its binding protein Fscn1. Therefore, we hypothesized that ANTXR1 can bind directly to the cytoskeleton and regulate the expression of Actin or stress fiber reorganization, thus transferring mechanical signals into the cell. However, the western blotting results in this study showed that the downregulation of ANTXR1 did not affect the expression levels of Actin and Fscn1. As the antibody in this work recognizes filamentous actin (F-actin) and reportedly also recognizes globular actin (G-actin)., and the actin molecules solubilized should be mostly G-actin, we inferred that the negative result of Actin expression illustrated in Fig. 7a could be attributed to G-actin concealing changes in F-actin because F-actin but not G-actin has been proven to be a stress-sensitive molecule. Although we predict that the F-actin/G-actin ratio is very likely to increase in pressure-promoted BMSCs, further detailed study is needed. Afterwards, the relationship between ANTXR1 and F-actin will require further exploration to examine the ratio of F-actin to G-actin, F-actin turnover, polymerization, etc.

Chondrogenic differentiation of BMSCs can be potently induced by TGF-β^52–54^. It was found that Smad2/4 could bind to the promoter of Fscn1α and that F-actin and Fscn1 are essential for activating the Nodal/Smad2 signaling pathway and for endodermal formation in the TGF-β family^55,56^. In this work, as we have found that ANTXR1 can interact with Actin and Fscn1, we further attempted to investigate the relationship between ANTXR1 and Smad2/3/4, which were reported to have close relationships with both Fascin1 and chondrogenesis^37–40^. We confirmed that hydrostatic pressure can activate the phosphorylation of Smad2 and the expression of Smad4. More importantly, inhibition experiments showed that Smad2 phosphorylation under hydrostatic pressure was completely ANTXR1-dependent. In addition, the results of Co-IP experiments showed that ANTXR1 was not directly associated with Smad2, P-Smad2, Smad3, P-Smad3, or Smad4. Smads 2 and 3 are transcription factors; once phosphorylated, they form hetero-oligomeric complexes with the transcription factor Smad4. These complexes enter the nucleus, bind promoters, and regulate chondrogenic target gene expression^57,58^. Here, we deduced that Smads are likely the downstream molecules of ANTXR1, and the mechanotransduction initiated by ANTXR1 could specifically activate Smad2 and up-regulate smad4 expression to facilitate the transport of activated smad2 to the nucleus to regulate chondrogenesis, which may be involved in the regulation of actin/fascin1. Unexpectedly, this work found that Smad3 did not participate in the chondrogenesis response and was not affected by the downregulation of either ANTXR1 or integrinβ1, although many other studies showed that Smad3 is involved in enhancing the transcriptional activity of Sox-9, a master regulator of chondrogenesis, in human MSCs^59–61^. This inconsistency is likely related to the specific mechanical stimulation used in this study, and the results remain to be confirmed by further research.

The intracellular biomechanical signal transduction system is complex, and integrinβ1 plays an important regulatory role in the mediation of mechanically stimulated chondrocyte differentiation and cartilage matrix formation^62–65^. When we focused on the mechanical pressure receptors in BMSCs, we noticed that although integrins are most often discussed as the cell’s primary mechanoreceptor on the cell membrane, several non-integrin mechanoreceptors have emerged over the last decade^66^. ANTXR1 is a membrane receptor, and its structural domains are similar to those of integrinβ1. ANTXR1 can function as an integrin in the following ways: 1) its extracellular domain can interact with type I and type VI collagen and gelatin, and the tail of its cytoplasmic portion can directly anchor to the actin cytoskeleton, assisting with cell adhesion and stretch and regulating cell expansion by coupling extracellular matrix ligands with the intracellular cytoskeletal system^60,67,68^; and 2) ANTXR1 has a high structural similarity with the integrin α subunit, which may allow it to function as an integrin, with its extracellular segment binding to the extracellular matrix components as endogenous ligands^27,69,70^. Garlick proposed that because the ANTXR1 protein is similar to integrins, despite the fact that they are independent of each other, and ANTXR1 has such a close relationship with the cytoskeletal system, Determining whether ANTXR1 is a new type of mechanical signal transduction molecule is worthy of further investigation^71^. In our experiments, ANTXR1 and integrinβ1 were used as the primary research subjects to perform a series of controlled studies. In BMSCs, both molecules were able to respond to stress stimuli and to participate in the process of pressure-promoted chondrogenic differentiation. Further studies examining the interaction between ANTXR1 and integrinβ1 in BMSC sheets showed that an interaction existed between ANTXR1 and integrinβ1, regardless of whether BMSC sheets were subjected to pressure stimulation; however, the inhibition experiments also confirmed that the functions of ANTXR1 and integrinβ1 were independent to each other. The results of the entire study suggested that both membrane signal-sensing molecules, ANTXR1 and integrinβ1, can independently respond to mechanical signals. Further examination of the downstream signaling molecules of ANTXR1 found that ANTXR1 could specifically activate Smad2 and up-regulate smad4 expression to facilitate the transport of activated smad2 to the nucleus to regulate chondrogenesis, which might be involved in the regulation of actin/fascin1. In addition, the classical wnt signaling pathway was partially regulated by ANTXR1 to transfer mechanical signals into the nucleus through β-catenin, which in turn promoted the chondrogenesis of BMSCs by upregulating the Sox-9, aggrecan and Col-II genes.

## Methods

### BMSC isolation and culture

Male, Sprague-Dawley (SD), 2-week old rats were obtained from the Laboratory Animal Center of the Fourth Military Medical University (Xi’an, China). Rat BMSCs (rBMSCs) were isolated and cultured according to the protocol reported by Maniatopoulos^72^. Briefly, bone marrow from femoral and tibial bones was aspirated with 12 mL of α-minimal essential medium (α-MEM, Corning Cellgro, USA), supplemented with 10% fetal bovine serum (FBS, Hangzhou Sijiqing Biological Engineering Materials Co., Ltd. China) and 1% antibiotic-penicillin/streptomycin (Sigma Aldrich, USA). The cells were incubated at 37 °C in a humidified atmosphere of 5% CO_2_ and 95% air until cells grew out from the tissue pieces. After 48 h, non-adherent cells were discarded, and adherent cells were thoroughly washed twice with phosphate-buffered saline (PBS, Corning Cellgro, USA). Fresh complete medium was added and replaced every 2 days for approximately 7 days. The primary culture cells were then subcultured, using a limiting dilution technique, to obtain passage 0 single-cell-derived clones (P0). All methods used in this study were performed in accordance with the approved guidelines and regulations of the Fourth Military Medical University (Xi’an, China). This study was approved by the Committee on the Ethics of Animal Research of the Fourth Military Medical University. All surgeries were performed under pentobarbital sodium anesthesia, and every effort was made to minimize the suffering of the animals.

### Flow cytometric analysis of cell surface markers

To characterize the immunophenotypes of rBMSCs, flow cytometric analysis was used to measure the expression of mesenchymal stem cell (MSC) and non-MSC-associated surface markers at early passages (P2). Briefly, adherent cells were washed twice with PBS and liberated by the addition of 2 mL 0.05% trypsin (Sigma, USA). Then, the single-cell suspension was washed twice and resuspended in PBS containing 3% FBS. To identify the MSC phenotypes, approximately 5 × 10^5^ rBMSCs/200 µL of PBS were placed in Eppendorf (EP) tubes and incubated with phycoerythrin (PE)- or fluorescein isothiocyanate (FITC)-conjugated monoclonal antibodies against rat CD44, CD90 and CD45 (BD Biosciences, USA) at 4 °C, in the dark. Samples incubated without antibodies were used as negative controls. After 1 h, the cells were washed twice with 1 mL wash buffer. Finally, labeled cells were analyzed using a flow cytometer (Beckman Coulter, USA).

### Osteogenic/adipogenic differentiation of BMSCs

To determine the multiple differentiation capacities of rBMSCs, 2 × 10^5^ rBMSCs (P3) were cultured with α-MEM in 6-well plates, without inducers, until confluence. At confluence, the medium was changed to either osteogenic medium or adipogenic medium. The osteogenic medium supplemented with 50 µg/mL L-ascorbic-2-phosphate (MP Biomedicals, USA), 0.1 mM dexamethasone, and 5 mM β-glycerophosphate (Sigma Aldrich, USA) in basal medium. The adipogenic medium supplemented with 1 µM dexamethasone, 10 mM insulin, 0.5 mM 1-methyl-3-isobutylxanthine (IBMX), and 200 µM indomethacin (Sigma Aldrich, USA) in basal medium. The induction medium was refreshed at 3-day intervals. For osteogenic induction, the cells were fixed with 4% PFA after 4 weeks of culture and stained with 2% Alizarin Red S (pH 4.2) (Kermel, China) and an alkaline phosphatase (ALP) color development kit (Beyotime, China). For adipogenic induction, the cells were fixed with 4% paraformaldehyde (PFA) after 3 weeks of culture and stained with 0.3% Oil Red O (Sigma Aldrich, USA), and lipid droplets were identified microscopically. Unbound and nonspecifically bound stain was removed by copious rinsing with distilled water, and stained calcium nodules or blue metachromatic regions were identified microscopically. Uninduced control cells were negative for Alizarin Red S, Oil Red O, and ALP staining.

### BMSC sheets induction

Cell cultures P1 were used for lentiviral transfection, and cell cultures at P3 were used for contrastive investigation in the present study. The procedures for the engineering of BMSC sheets and lentiviral transfection can be found in the Supplementary Materials. BMSCs (P3) were plated on 6-well plates, at a density of 3 × 10^5^ cells/well, and cultured for 24 h to allow the cells to reach 80% confluence. Then, the cell culture medium was replaced with cell sheet-inducing medium, α-MEM supplemented with 10% FBS, 1% penicillin and streptomycin and 50 µg/mL L-ascorbic acid (Vitamin C, Sigma, USA). Sheets began to form after 2 weeks of culture.

### Cytomechanical loading strategy using hydrostatic pressure on BMSCs

To simulate compressive stress on cultured BMSCs, we applied a new multi-functional hydrostatic cellular pressure unit (see Supplementary information). The system consisted of three parts: a cell culture system, a loading control system, and a data processing system (Fig. S6). Different modes of pressure could be applied to the cells, and a series of biological effects in stem cells were further evaluated. The parameter settings were as follows: the pressure ranged from −50 to 300 kPa, the accuracy of dynamic pressure was controlled within ±5%, the accuracy of static pressure was controlled within ±1% for negative pressure or ±3% for compressive pressure, the temperature was 36 ± 2 °C, and the frequency of load ranged from 0.01 Hz to 0.1 Hz. This device could overcome temperature compensation caused by different types and different ranges of pressure by using a combination of a thermostatic water bath and an auxiliary heating device to maintain a constant temperature for cell cultures. This device could provide a relatively large range of pressure, using a combined loading system, and could monitor every change in pressure and temperature inside the incubator, in real time, by using monitoring software (Fig. S6). Additionally, this system is easy to handle, precise and stable and has multiple pressure modes and reliable performance, making it suitable for research on other types of stress-sensitive cells (e.g., articular chondrocytes, osteoblast, and periodontal ligament cells).

### Selection of favorable hydrostatic pressure conditions for ANTXR1 reactions and BMSC chondrogenesis

In the present study, the BMSC sheets were stimulated with hydrostatic pressure, using the above-mentioned, multi-functional pressure unit. Our group has demonstrated that, in an environment loaded with pressure using the compression device, 90 kPa for 1 h can result in extensive biological effects on BMSCs, including enhanced cell proliferation activity, elevated ALP activity, the upregulation of estrogen receptor-α expression, and the assembly of intracellular stress fibers^13,14^. In the present study, we compressed BMSC sheets, rather than monolayer-cultured BMSCs. Therefore, based on the optimal pressure condition for BMSCs that were used in previous experiments, we increased the force value to further determine the most suitable conditions of pressure stimulation for the chondrogenic differentiation of BMSC sheets. During the experiment, 6-well plates containing the cell sheets were placed in the cell compression device and stimulated with pressure under fixed conditions for 1 h. Biomechanical treatment groups received either static pressure, including 0 kPa, 90 kPa, 120 kPa, 150 kPa, and 180 kPa, or dynamic pressure, including 0–90 kPa, 0–120 kPa, 0–150 kPa, and 0–180 kPa. Afterwards, the effects of the hydrostatic stimulation on BMSCs were evaluated.

The relative mRNA expression levels of ANTXR1, integrinβ1, Sox-9, aggrecan, and Col-II (GeneCopoeia, USA) in BMSC sheets were determined immediately following the 14-day cell sheets induction. Total RNA from BMSC sheets were isolated using TRIzol reagent (Invitrogen, USA). Then, 2–5 µg of total RNA was converted into cDNA, using a Revert Aid First Strand cDNA Synthesis Kit (Takara, Japan). Real-time polymerase chain reaction (RT-PCR) was performed using SYBR® Premix Ex Taq™ II kit (Takara, Japan) in a quantitative PCR System (Bio-Rad, USA). Amplification was performed under the following conditions: denaturation at 95 °C for 3 min, followed by 39 cycles at 95 °C for 15 s and 60 °C for 30 s. The primers used in the present study are listed in Supplementary Table S5; glyceraldehyde 3-phosphate dehydrogenase (GAPDH) primers were used to normalize samples. The results were evaluated by the Smart Cycler II software program. All examinations were conducted in triplicate for each cell sheets.

Total proteins were extracted from the BMSC sheets by lysing in radioimmunoprecipitation assay (RIPA) buffer supplemented with a protease inhibitor cocktail (Sigma, USA). The protein concentration was determined using a bicinchoninic acid (BCA) protein assay kit (Beyotime, China). A total of 20 µg protein was added to each well of a Tris glycine sodium dodecyl sulfate (SDS)-polyacrylamide gel (Invitrogen), separated, and transferred onto a polyvinylidene fluoride (PVDF) membrane (Millipore, USA), followed by blocking with 5% bovine serum albumin (BSA) for 2 h. The membrane was probed overnight at 4 °C with primary antibodies for rat ANTXR1 (1:2,000, Abcam, USA), integrinβ1 (1:200, Santa Cruz, USA), Sox-9 (1:200, Novus, USA), aggrecan (1:100, Novus, USA), Col-II (1:200, Novus, USA), and GAPDH (1:4,000, Cowin Biotech, China). Then, membranes were incubated with secondary antibody at room temperature for 2 h. Signals were developed on film by exposing the membrane to a chemiluminescent horseradish peroxidase (HRP) substrate (Thermo Scientific Inc., USA) using the Western-Light Chemiluminescent Detection System (Peiqing, China). The antibodies used in the present study are listed in Supplementary Table S6. The gray values of the blots in the pictures were measured with ImageJ software. The gray value of each target protein was normalized to that of GAPDH before comparisons.

### Roles of ANTXR1 and integrinβ1 in the hydrostatic pressure-induced chondrogenesis of BMSC sheets

Integrinβ1 short-hairpin RNA (shRNA) and a scrambled negative control were synthesized and cloned into the lentiviral GV248 vector (Genechem, China). Lentivirus preparation, infection and selection were performed by the Genechem company. ANTXR1 shRNA was synthesized and cloned into the lentiviral psi-LVRU6GP vector (GeneCopoeia, USA). Lentivirus preparation, infection and selection were performed by the GeneCopoeia company. The targeted sequences are listed in the Supplementary Table S5. Rat BMSCs at passage P1 were placed in a 24-well plate, at a density of 5 × 10^4^ cells/well, treated with lentiviruses at different multiplicities of infection (MOI) for 72 h, and were then observed under either a fluorescent inverted microscope or a laser scanning confocal microscope (LSCM) to estimate the infection efficiency of the lentiviruses in BMSCs (see Supplementary Figs S8, S9). Then the cells were cultured and passaged to the P3 generation. Cell sheets formation was induced by incubation in sheet-inducing solution for 14 days to generate BMSC cell sheets containing lentiviruses. Subsequently, the expression levels of the target genes and proteins were detected by RT-PCR and western blotting to evaluate the transfection efficiency.

The cells were divided into the following six groups: blank control group, simple pressure group, ANTXR1-downregulated group, integrinβ1-downregulated group, ANTXR1-downregulated hydrostatic-pressure-stimulated group, and integrinβ1-downregulated hydrostatic-pressure-stimulated group. Gene and protein expression levels of chondrogenic markers in each group, including Sox-9, aggrecan and Col-II, were detected by RT-PCR and western blotting assays, with GAPDH as the reference molecule. The detailed RT-PCR and western blotting methods have been described.

### Relationship between ANTXR1 and integrinβ1

Antibodies against ANTXR1 (10 µL, Abcam, USA), integrinβ1 (10 µL, Santa Cruz, USA), and IgG (10 µL, Cell Signaling, USA) were subjected to clean-up using the Pierce™ Antibody Clean-up Kit (Thermo Scientific, USA). The clean-up was performed according to the manufacturer’s protocol. Total proteins from 3 sets of 6-well plates containing rat BMSC sheets were collected, combined, and used for co-immunoprecipitation (Co-IP) experiments performed with the Pierce™ Co-IP Kit (Thermo Scientific, USA). The Co-IP experiments were performed according to the manufacturer’s protocol. Subsequently, western blotting analysis of ANTXR1 and integrinβ1 proteins was performed.

For immunofluorescence (IF) assays, rat BMSCs were seeded into the wells of glass-bottomed dishes (Nest, USA) and incubated for 24 h. Next, the cells were fixed with 4% PFA for 20 min, after being rinsed twice with ice-cold PBS. The following primary antibodies were used: ANTXR1 (1:50, Abcam, USA) and integrinβ1 (1:10, Santa Cruz, USA). The cells were probed overnight at 4 °C. The following secondary antibodies were used: Cy3-Affini pure goat anti-rabbit IgG (1:200, Jackson, USA) and goat anti-mouse IgG (1:200, Jackson, USA). The cell nuclei were stained with Hoechst (1:50, Invitrogen, USA), and the cytoskeleton was stained with phalloidin (1:20, Sigma-Aldrich, USA), prior to LSCM imaging on a FV1000 microscope (Olympus, Japan).

After treatment, total cellular RNA was isolated from cell cultures by TRIzol (Invitrogen, Carlsbad, USA) to evaluate the expression levels of ANTXR1 and integrinβ1 by RT-PCR (TaKaRa Bio, Tokyo, Japan). The primer sequences for ANTXR1 and integrinβ1 (Sango Biotech, Shanghai, China) are listed in the Supplementary Materials, Table S5. The reaction products were quantified by using a relative quantification tool (CFX Manager, Bio-Rad), with GAPDH as the reference gene. The reactions were performed under the following conditions: 95 °C for 3 min, 39 cycles at 95 °C for 10 s and 60 °C for 35 s, and a melting curve from 60 to 95 °C, at increments of 0.5 °C, for 5 s. ANTXR1 and integrinβ1 protein expression levels were detected by western blotting assay, with GAPDH as the reference molecule. The membrane was probed overnight at 4 °C with primary antibodies for rat ANTXR1 (1:2,000, Abcam, USA) and Integrin β1 (1:200, Santa Cruz, USA).

### Detection of ANTXR1 downstream signaling molecules

Total proteins were extracted from BMSC sheets by lysing in RIPA buffer containing a protease inhibitor cocktail (Sigma). The protein concentrations were determined by a BCA protein assay kit (Beyotime, China). A total of 20 µg protein was added to each well of a Tris glycine SDS-polyacrylamide gel (Invitrogen), separated, and transferred onto a PVDF membrane (Millipore, USA), followed by blocking with 5% BSA for 2 h. The membrane was probed overnight at 4 °C with primary antibodies for rat LRP5 (1:1,000, Cell Signaling, USA), LRP6 (1:4,000, Abcam, USA), Actin (1:200, Abcam, USA), Fscn1 (1:20,000, Abcam, USA), Smad2 (1:1,000, Cell Signaling, USA), P-Smad2 (1:1,000, Cell Signaling, USA), Smad3 (1:1,000, Cell Signaling, USA), P-Smad3 (1:1,000, Cell Signaling, USA), Smad4 (1:1,000, Cell Signaling, USA), GSK3β (1:1,000, Cell Signaling, USA), P-GSK3β (1:1,000, Cell Signaling, USA), β-Catenin (1:1,000, Cell Signaling, USA) and Active β-Catenin (1:1,000, Millipore, USA). The antibodies used in the present study are listed in Supplementary Table S6.

Antibodies against ANTXR1 (10 µL, Abcam, USA) and IgG (10 µL, Cell Signaling, USA) were subjected to clean-up using the Pierce™ Antibody Clean-up Kit (Thermo Scientific, USA). The clean-up was performed according to the manufacturer’s protocol. Total proteins from 3 sets of 6-well plates containing rat BMSC sheets were collected, combined, and used for immunoprecipitation (IP) experiments, performed using the Pierce™ Co-IP Kit (Thermo Scientific, USA) according to the manufacturer’s protocol. Subsequently, western blotting analysis of LRP5, LRP6, Actin, Fscn1, Smad2, P-Smad2, Smad3, P-Smad3, Smad4, GSK3β, P-GSK3β, β-Catenin and active β-Catenin protein expression was performed.

### Data analysis

All data are expressed as the mean ± standard deviation from at least three independent experiments. The data were analyzed using a one-way analysis of variance (ANOVA), combined with Student-Newman-Keuls post hoc test, or Student’s t-test, using SPSS 19.0 software (SPSS, USA). A *p* value of < 0.05 was considered to be significant.

## Supplementary information

Supplementary materialsL
