# Supplementary material for: The role of anthrax toxin protein receptor 1 as a new mechanosensor molecule and its mechanotransduction in BMSCs under hydrostatic pressure
Source: Sci Rep. 2019 Sep 2;9:12642. doi: 10.1038/s41598-019-49100-5 (PMC6718418; doi:10.1038/s41598-019-49100-5)
Supplement: Supplementary file 1 — Supplementary materialsL [file 41598_2019_49100_MOESM1_ESM.doc]

**Supplementary Information for**

**The role of anthrax toxin protein receptor 1 as a new mechanosensor molecule and its mechanotransduction in BMSCs under hydrostatic pressure**

Baixiang Cheng^1^, Yanzheng Liu^1^, Ying Zhao^1^, Qiang Li, Yanli Liu, Junjun Wang, Yongjin Chen*, Min Zhang*

State Key Laboratory of Military Stomatology & National Clinical Research Center for Oral Diseases & Shaanxi International Joint Research Center for Oral Diseases, Department of General Dentistry and Emergency, School of Stomatology, Fourth Military Medical University, No.145 West Changle Road, Xi'an, 710032, China

^1^ These authors contributed equally to this article.

*Corresponding author: Min Zhang, *Co-corresponding author: Yongjin Chen

State Key Laboratory of Military Stomatology & National Clinical Research Center for Oral Diseases & Shaanxi International Joint Research Center for Oral Diseases, Department of General Dentistry and Emergency, School of Stomatology, Fourth Military Medical University, Xi'an, China

Tel.: +86 29 84772538

1. mail: [zhangmin@fmmu.edu.cn](mailto:zhangmin@fmmu.edu.cn), cyj1229@fmmu.edu.cn

**This PDF file includes ADDITIONAL INFORMATIONS and SUPPLEMENTARY FIGURES, TABLES and REFERENCES.**

**Supplementary Information**

**ADDITIONAL METHODS AND INFORMATION**

**Differential protein expression analysis in BMSCs treated with or without hydrostatic pressure stimulation by SILAC**

Our preliminary studies found that signaling pathways in BMSCs, such as bone morphogenetic protein (BMP)/Smad, p38/nuclear factor (NF)-κB, Wnt/β-catenin, and Wnt/Ca^2+^, are involved in the mechanical signal transduction processes in BMSCs (unpublished data). However, the exact mechanisms through which BMSCs sense mechanical stimulation at the cell membrane level and initiate the mechanical signal transduction process are still unclear. To better understand the mechanical and biological responses to stress and the signal transduction mechanisms in BMSCs, our team used the stable isotope labeling with amino acids in cell culture (SILAC) method to identify proteins with differential expression levels before after mechanical stimulation. SILAC is an effective method for the identification and quantification of proteins with differential changes in expression levels in cultured cells. The "heavy" 13C- or 15N-labeled amino acids are incorporated into proteins, allowing the proteins to be rapidly and comprehensively identified, characterized and quantified by mass spectrometry (MS). Using this method, 48.83% of proteins (2,142 proteins) in BMSCs were found to have differential expression levels after treatment with 120 kPa of hydrostatic pressure for 1 h, of which 37.44% were upregulated and 11.39% were downregulated (Fig. S1). Then, we used Kyoto Encyclopedia of Genes and Genomes (KEGG) and gene ontology (GO) software to perform an informatics analysis of the differentially expressed proteins. This in-depth study of the stress-stimulated membrane-sensing signaling molecules in BMSCs revealed that the expression of integrin, a classical membrane mechanosensor molecule in BMSCs, was upregulated. However, when examining a series of signaling pathways closely related to membrane mechanical sensitivity, such as cell leading, , actin cytoskeleton and actin binding, the most significant changes were identified for the same molecule, anthrax toxin receptor 1, ANTXR1 or tumor endothelial Marker 8, TEM8.(Table S2-S4).





**Figure S1.** Differentially expressed proteins by SILAC in BMSCs before and after 120 kPa of hydrostatic pressure was applied for 1 h. (a) Schematic diagram of double-SILAC detection for BMSCs before and after the pressure stimulation. (b) At total of 2,142 differentially expressed proteins were detected by SILAC analysis. (c) Analysis showed that 37.44% of differentially expressed proteins were upregulated and 11.39% were downregulated. (d) Total protein network map (red represents upregulated proteins, green represents downregulated proteins; the darker the color, the greater the change).

**Cytomechanical loading strategy of applying hydrostatic pressure to BMSCs.**

In cell biomechanics studies, the biological similarity of mechanical stimulation has great implications for the reliability of the study results and further applications in the future. Generally, mandibular condylar cartilage suffers pressure from frequent jaw movement and biting hard food ^1^, although it is not possible to determine the real mechanical load that the condylar cartilage endures *in vivo*. In our previous work, the results of finite element analysis showed that the stress on the condylar cartilage was compressive under normal occlusion ^2^. In articular cartilage, collagen forms a three-dimensional network and thus affects the form, stability, tensile strength and resistance of the cartilage to external stresses and strains^3^. When cartilage is loaded by compression, the low permeability of the collagen network impedes the flow of interstitial fluids through the collagen network ^4^. Thus, the cartilage tissue does not significantly deform under these conditions, and any potential interference caused by cartilage deformation is prevented. Therefore, during mandibular movement, the pressure applied to the mandibular condyle across the teeth and mandible is similar to hydrostatic pressure applied to the condylar chondrocytes^5^.

***Background of the present research work and the cyto-mechanical environment design.***

As our series of research is dedicated to examined the regeneration and repair of the stress-sensitive cartilage of the temporomandibular joint BMSCs. First, the biomechanical characteristics of articular cartilage in vivo were explored. Then, the mechanobiological effects of the chondrogenic differentiation of BMSCs and its mechanotransduction mechanisms under a simulated biomechanical environment were studied. Ultimately, we determined that the mechanisms involved in the regulation of BMSC differentiation occurred through the up or down-regulation of specific mechanotransduction molecules. It is not possible to determine the real mechanical load that the condylar cartilage endures in vivo. In our previous work, the results of finite element analysis showed that the stress in the condylar cartilage was compressive under a normal occlusion (Fig. S2). In articular cartilage, collagen forms a three-dimensional network and thus affects the form, stability and tensile strength and resistance of the cartilage to external stresses and strains. When a cartilage is loaded by compression, the low permeability of the collagen network impedes the interstitial fluid to flow through the collagen network. Thus, the cartilage tissue did not significantly deform under these conditions. Thus, any potential interference caused by cartilage deformation was prevented. Therefore, during mandibular movement, the pressure applied to the articular cartilage across the teeth and mandible was similar to hydrostatic pressure on deep tissue. As such, we applied a self-designed hydraulic pressure-controlling cellular strain unit that imitated the mechanical pressures of the chondrocytes or cartilage-targeted stem cells in vivo. Additionally, the in vitro cell culture model under the hydrostatic compression has several attractive properties: simplicity of the equipment, spatial homogeneity of the stimulus, ease of configuring multiple loading replicates (via manifolding), and ease of delivering and transducing either static or transient loading inputs. There were no physical impediments to the metabolite transport processes between the culture layer and the nutrient medium. Therefore, the mechnaotransductive nature of the chondrocytes or cartilage-targeted stem cells was much simpler to investigate.

***Selection of feasible pressure condition***

In our previous studies, the results of finite element analyses showed that the stress in the condylar cartilage of TMJ was approximately 300 kPa under normal occlusion. Therefore, in our previous studies on the pressure-induced mechanobiology of primary chondrocytes using a hydraulic pressure-controlling cellular strain unit, we limited the range of pressures on the cells from 30 to 300 kPa (as calculated value by the finite element method). The cells were divided into several testing groups and subjected to different pressures, i.e., 30, 60, 90, 120, 240, and 360 kPa. However, we found that under high pressures, the form and function of the cells were significantly affected. Because of this observation, we further narrowed the range of pressures below 100 kPa and re-grouped the cells as shown in Tab. S1. In the experiments studying the mechanotransduction of BMSCs adopted for cartilage regeneration, the same mechanical unit as those used in previous works was utilized and the feasible pressure condition for mandibular chondrocytes, 90 kPa/h, was also adopted. It was found that a 90 kPa/h pressure could effectively promote proliferation and the chondrogenic differentiation ability of the BMSCs. However, a long-term application of pressure (e.g., 6 h) or a relatively higher pressure stimulation (e.g., 120 kPa or 150 kPa for 1 h) were not as effective for BMSCs, PCNA and chondrogenic gene expressions (Fig. S3). We further investigated changes to the cytoskeletal structures of BMSCs under mechanical pressure. In cells subjected to 90 kPa for 1 h, thick filamentous structures formed and were densely aligned parallel to the long axes of the cells (Fig. S4). In contrast, the cytoskeleton of cells subjected to 90 kPa for 6 h showed broken and disordered filamentous structures. Additionally, in response to 90 kPa for 1 h, F-actin showed more observable stress fibers than those subjected to 90 kPa for 6 h (Fig. S5). As to the present study, we detected the mechantranduction of BMSCs in cell sheet, which will be used as transplant for further tissue-engineering cartilage regeneration, but not the BMSCs in monolayer as we have studied before. Therefore, the pressure conditions used in the experiments of this present study were re-screened as Fig. 2, and the feasible pressure condition was set at 120 kPa for 1h. I hope the detailed explanation provides a much clearer guideline for the present study. As the reviewer suggested, our future experiments will focus on verifying the in vivo regulatory effects of ANTXR1 on BMSCs for articular cartilage regeneration in TMJ of rabbits.
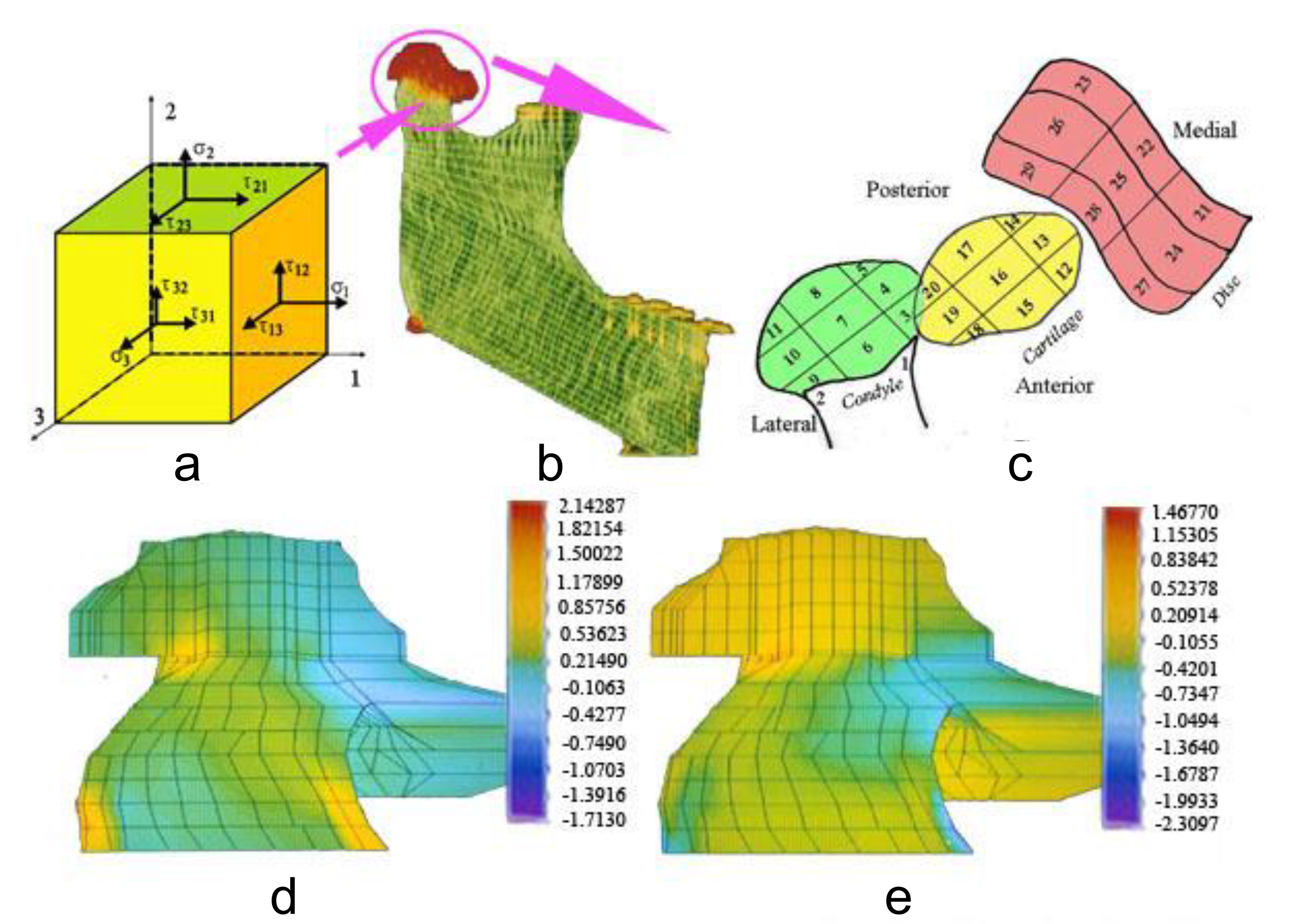


**Figure S2**. FEA results of TMJ stress. a: Three-dimensional finite element mesh of the TMJ, including the mandible, condyle, disc and cartilage. b: Three-dimensional finite element model of TMJ. c: 29 Observation points map for stress values collection. d: Distribution map of the maximal main stress in each part of TMJ. e: Distribution map of the minimal main stress in each part of TMJ.

**Table S1 ALP activity and cell proliferation under different pressure (χ±s)**

**Time (min) 0 Kpa 30 Kpa 60 Kpa 90Kpa**

**ALP**  **0 0.102±0.003 0.098±0.006 0.103±0.005 0.103±0.004**

**(n=8) 60 0.104±0.006 0.101±0.004 0.107±0.003 0.112±0.004***

**360 0.142±0.006 0.134±0.005 0.132±0.004* 0.203±0.015****

**720 0.183±0.005 0.176±0.002 0.212±0.004** 0.198±0.009****

**Proliferation 0**   **0.30±0.02 0.30±0.02 0.28±0.01 0.29±0.02**

**(n=6) 60 0.31±0.03 0.29±0.04 0.30±0.03 0.28±0.04**

**360 0.45±0.01 0.43±0.02 0.39±0.02** 0.37±0.01****

**720 0.62±0.02 0.57±0.03 ^a^ 0.42±0.02** 0.38±0.03****

* *vs* 0Kpa p<0.05 ** *vs* 0Kpa p<0.01


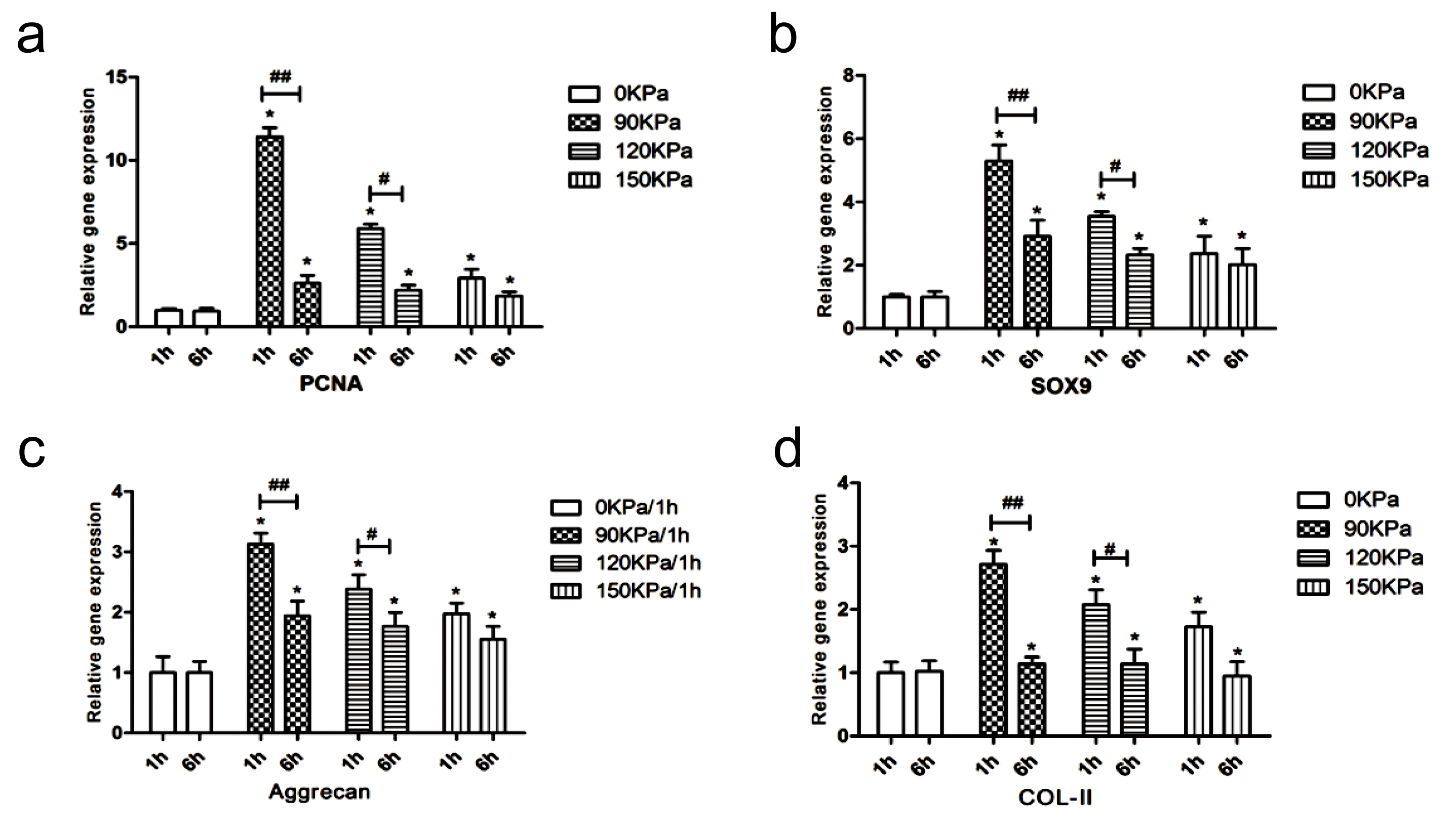


**Figure S3**. Real-time PCR assay of proliferation marker PCNA mRNA (a) and chondrogenic markers of Sox-9 (b), Aggrecan (c) and Col-II (d) mRNA expression in monolayer-cultured BMSCs treated with 90, 120, 150 Kpa hydrostatic pressure by different time .


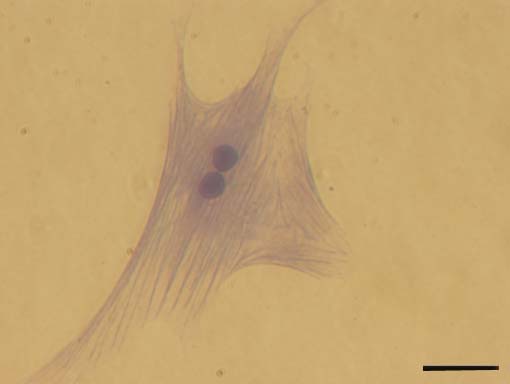

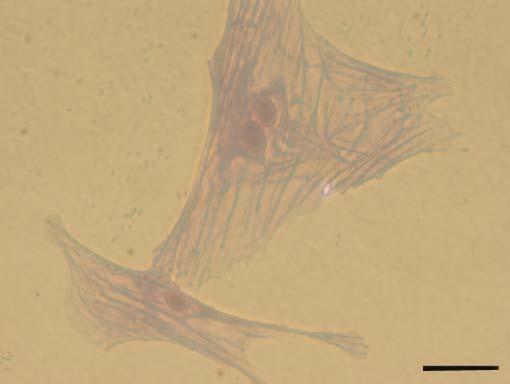

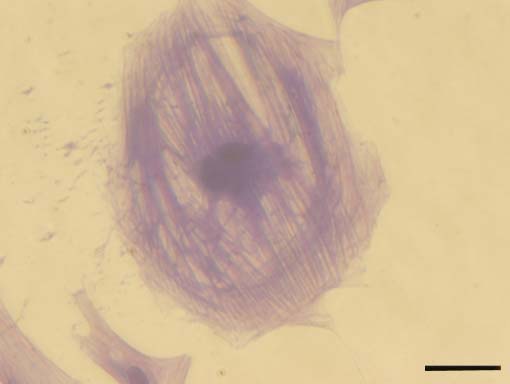

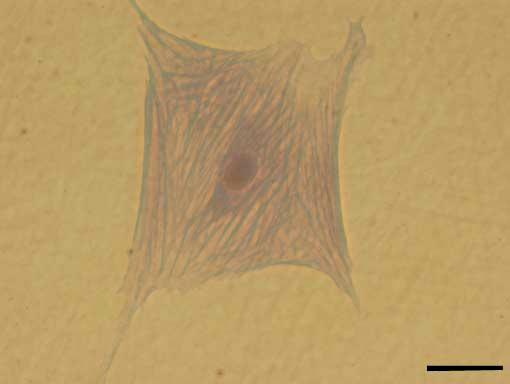


**Control**

**90 Kpa / 1 h**

**90 Kpa / 6 h**

**90 Kpa / 6 h**

**Figure S4**. Coomassie brilliant blue staining of the cytoskeleton of monolayer-cultured BMSCs under different pressure conditions.


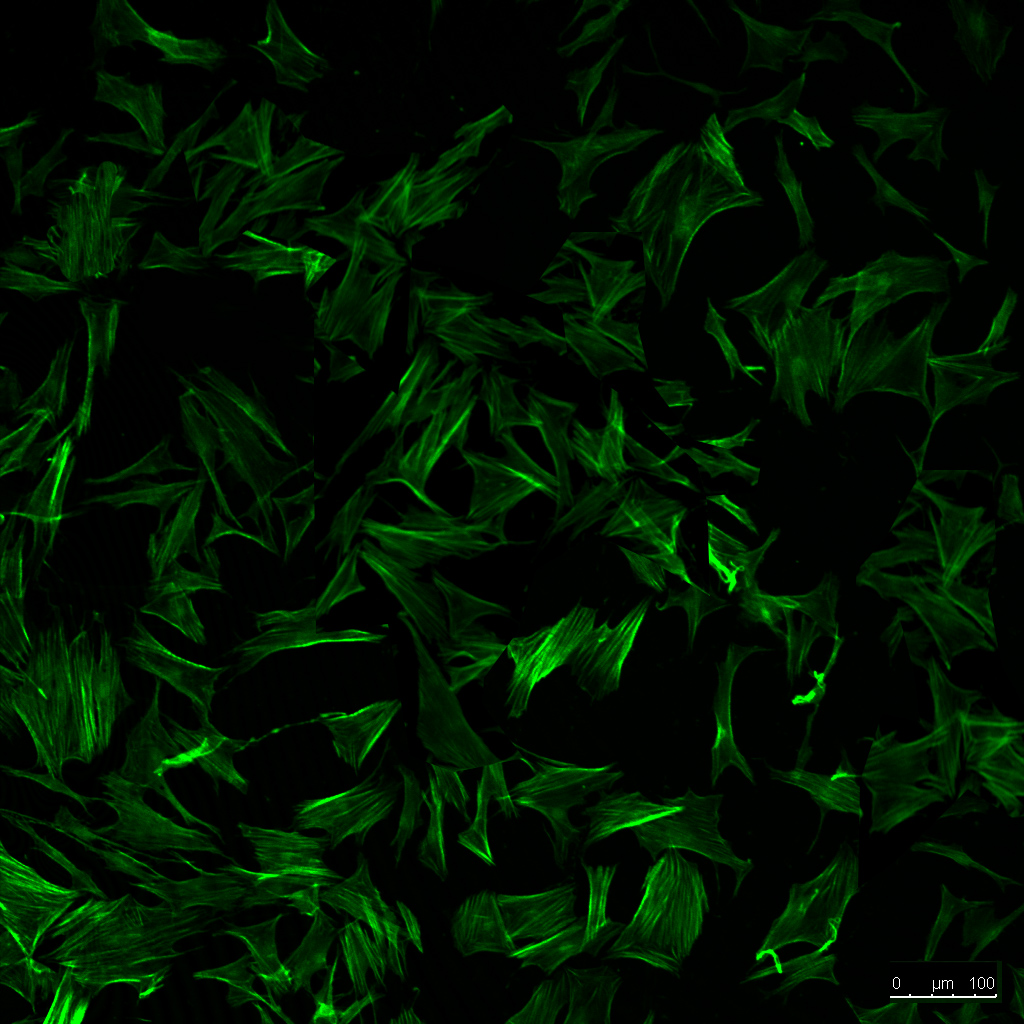

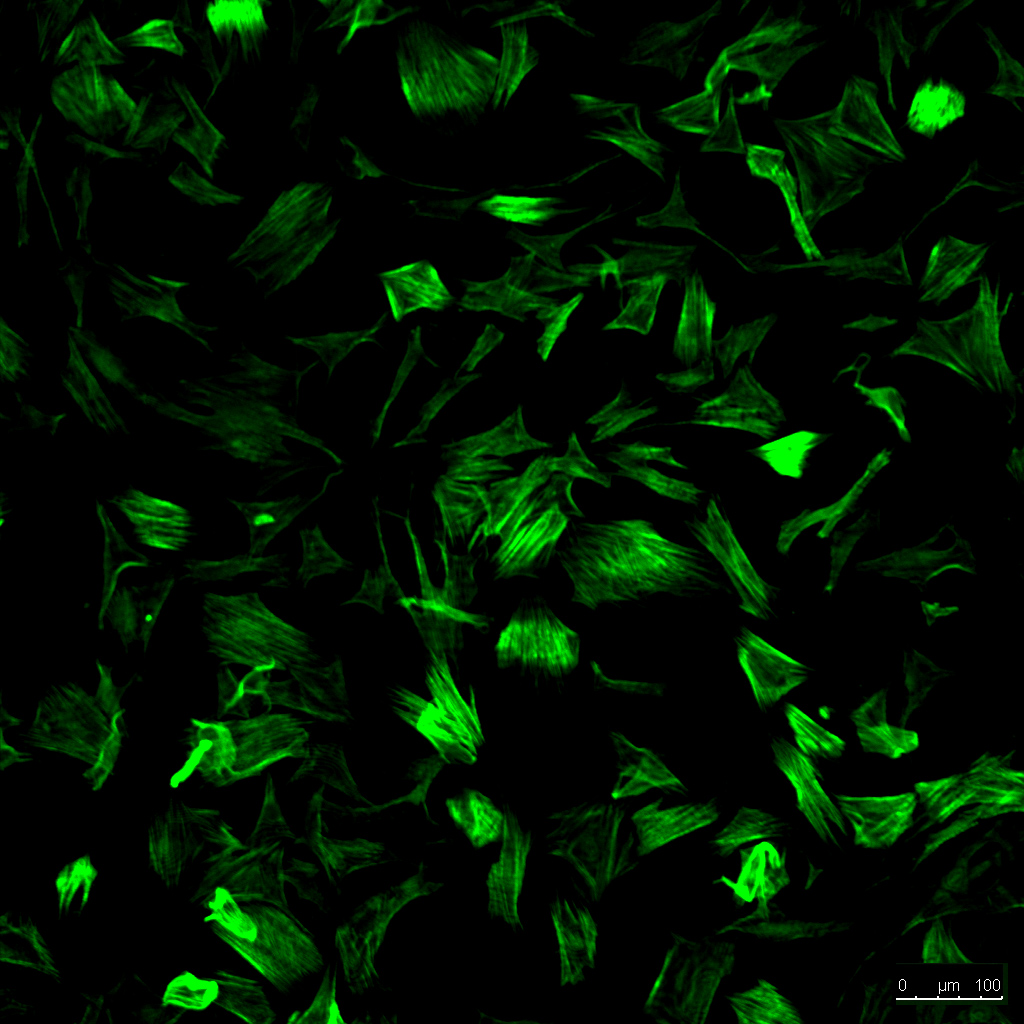

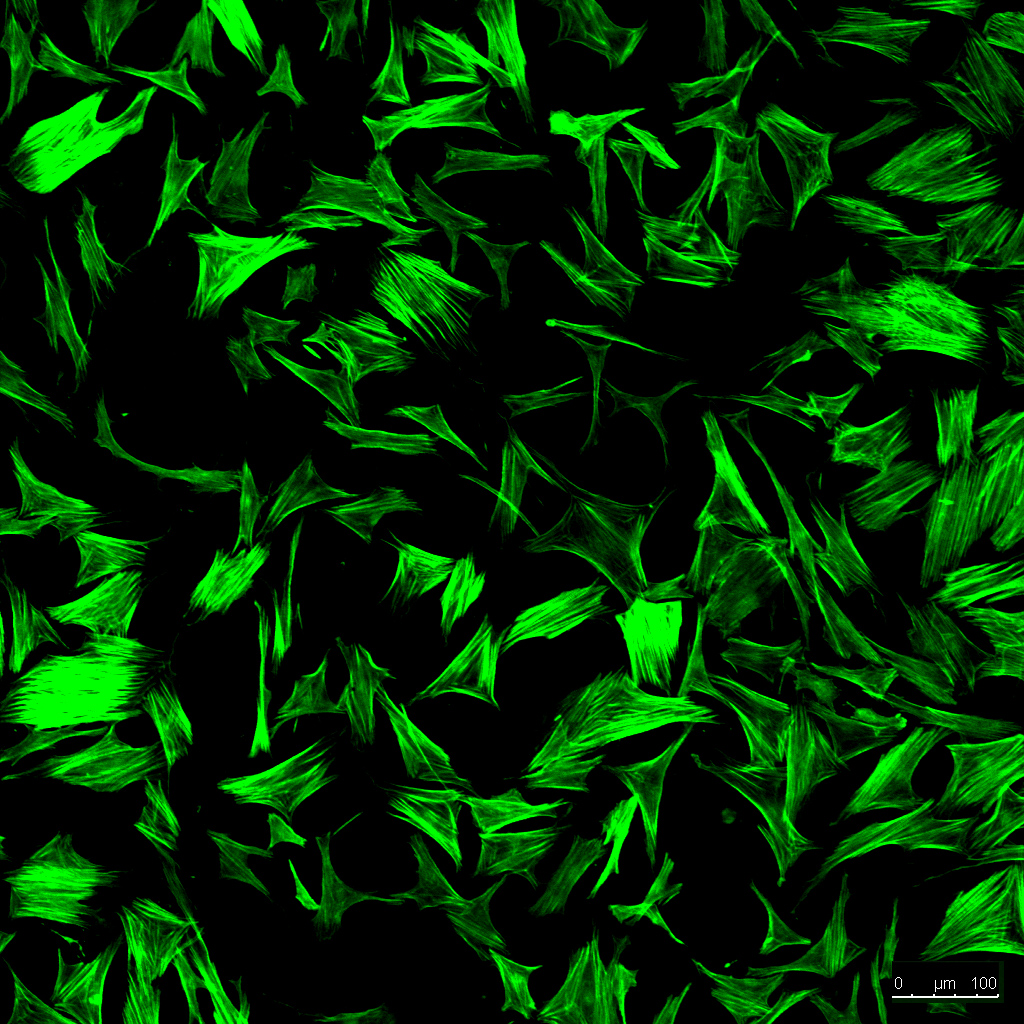


**Control**

**90 kPa / 1 h**

**90 kPa / 6 h**

**Figure S5**. FITC-phalloidin staining for F-actin in monolayer-cultured BMSCs under different pressure conditions.

***Introduction of the multi-functional hydrostatic cellular pressure unit***

Hydrostatic pressurization has been a frequently used modality for the compression of cells, tissues, or explant cultures and includes both negative (vacuum) and positive pressurization. Hydrostatic compression has several attractive properties: simplicity of the equipment, spatial homogeneity of the stimulus, ease of configuring multiple loading replicates (via manifolding), and ease of delivering and transducing either static or transient loading inputs. There is no physical impediment of the metabolite transport processes between the culture layer and the nutrient medium. Moreover, the load delivered does not depend on the state of adhesion between the culture and its substrate. The multi-functional hydrostatic cellular pressure unit adopted in the present study consisted of three parts: a cell culture system, a loading control system, and a data processing system (Fig. S6). Different modes of pressure could be applied to the cells, and a series of biological effects in stem cells were further evaluated. The parameter settings were as follows: the pressure ranged from -50 to 300 kPa, the accuracy of dynamic pressure was controlled within ±5%, the accuracy of static pressure was controlled within ±1% for negative pressure or ±3% for compressive pressure, the temperature was 36±2°C, and the frequency of load ranged from 0.01 Hz to 0.1 Hz. This device could overcome temperature compensation caused by different types and different ranges of pressure by using a combination of a thermostatic water bath and an auxiliary heating device to maintain the temperature for cell cultures. This device could provide a relatively large range of pressure, using a combined loading system, and could monitor every change of pressure and temperature inside the incubator, in real time, by using monitoring software. Additionally, this system is precise, stable and easy to handle, and has multiple pressure modes and reliable performance, making it suitable for research on other types of stress-sensitive cells (e.g., articular chondrocytes, osteoblast, and periodontal ligament cells). Therefore, we applied a self-designed hydrostatic pressure-controlling cellular strain unit that imitated the mechanical pressures experienced by chondrocytes or cartilage-targeted stem cells.


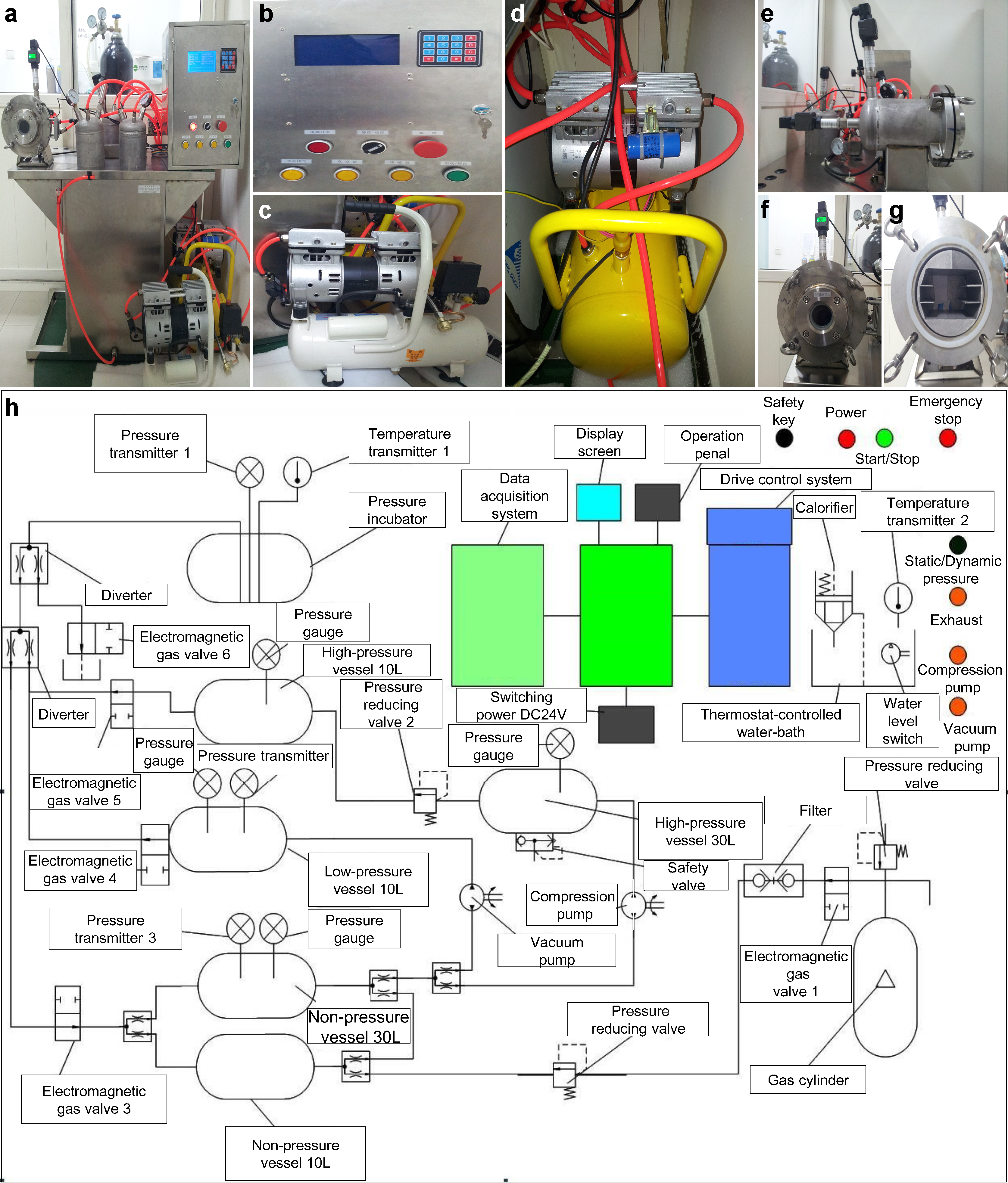


**Figure S6. The multi-functional pressure loading system for *in vitro* cultured cells.** (a) Overall view of the system. (b) Control panel. (c) Drive control system–compression pump. (d) Drive control system–vacuum pump. (e) Lateral view of the pressure incubator. (f) Front view of the pressure incubator. (g) Interior of the pressure incubator. (h) Schematic illustration of the whole system.

**Role of the Wnt/β-catenin signaling pathway in the chondrogenic differentiation of BMSCs under 120 kPa hydrostatic pressure**

In this study, the role of the Wnt/β-catenin signaling pathway in the chondrogenic response of BMSCs under 120 kPa hydrostatic pressure was observed using the classical Wnt signaling pathway blocker XAV939. The results of both a PCR assay and western blotting showed that hydrostatic pressure of 120 KPa for 1 h activated the expression of the cartilage-associated proteins Col-II, Sox-9 and Aggrecan in BMSCs. The expression of cartilage-associated genes and proteins in the BMSCs pretreated with the Wnt/β-catenin signaling pathway inhibitor XAV939 was significantly lower than in the treatment groups without XAV939 after the same biomechanical stimulation (P<0.05). This result indicated that the Wnt/β-catenin signaling pathway plays a positive role in the cartilage response of pressure-regulated chondrogenesis of BMSCs (Fig. S7)


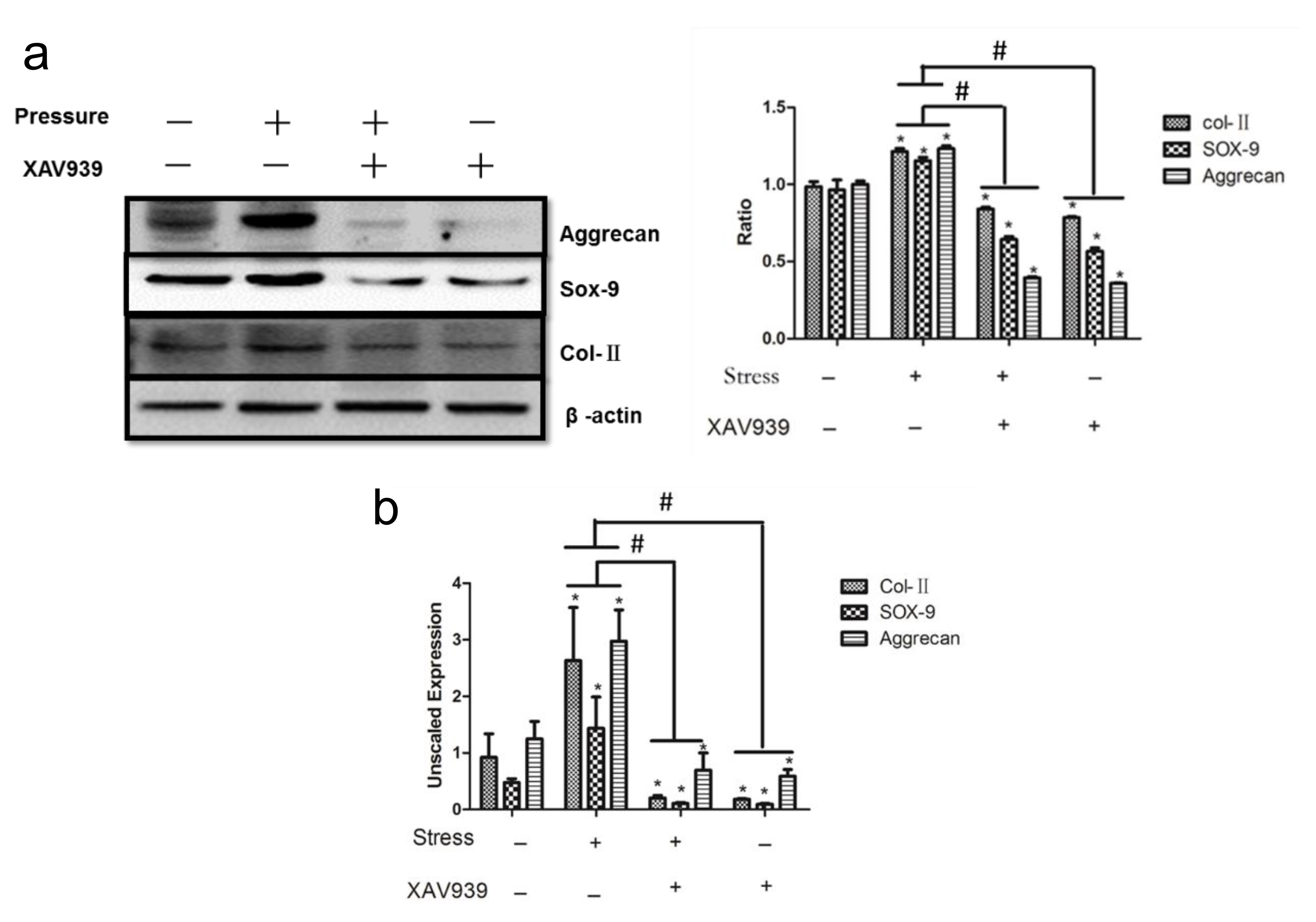


**Figure S7.** Expression of cartilage-related genes and proteins of Col-II, Sox-9 and Aggrecan in BMSCs under pressure of 120KPa for 1h (*P<0.05 versus Control and # P<0.05 versus the indicated groups)

**Lentivirus-transfected rat BMSC sheets**

Integrinβ1 short-hairpin RNA (shRNA) and a scrambled negative control were synthesized and cloned into the lentiviral GV248 vector (Genechem, China). Lentivirus preparation, infection and selection were performed by the Genechem company. ANTXR1 shRNA was synthesized and cloned into the lentiviral psi-LVRU6GP vector (GeneCopoeia, USA). Lentivirus preparation, infection and selection were performed by the GeneCopoeia company. The targeted sequences are listed in the Table S5. The packaged lentivirus was transfected into P1 generation rat BMSCs, and then the cells were cultured and passaged to the P3 generation. Cell sheet formation was induced by incubation in sheet-inducing solution for 14 days to generate BMSC cell sheets containing lentiviruses (Fig. S8). First, the pre-experiment of cell transfection was performed. Cells were transfected with the lentivirus, using multiple MOI values, for 72 hours. When cells transfected at MOI=40 was observed by fluorescence microscopy, the lentiviral transfection efficiency was above 80%, and the cells did not show signs of lysis or death. Next, the cells were passed to the P3 generation and induced to form a cell sheet. Under a fluorescence microscope, the cell transfection efficiency was determined to be above 80%, and the state of the cell sheet was the same as that of a normal cell sheet, indicating that we can successfully construct cell sheets using rat BMSCs transfected with lentiviruses (Fig. S9).


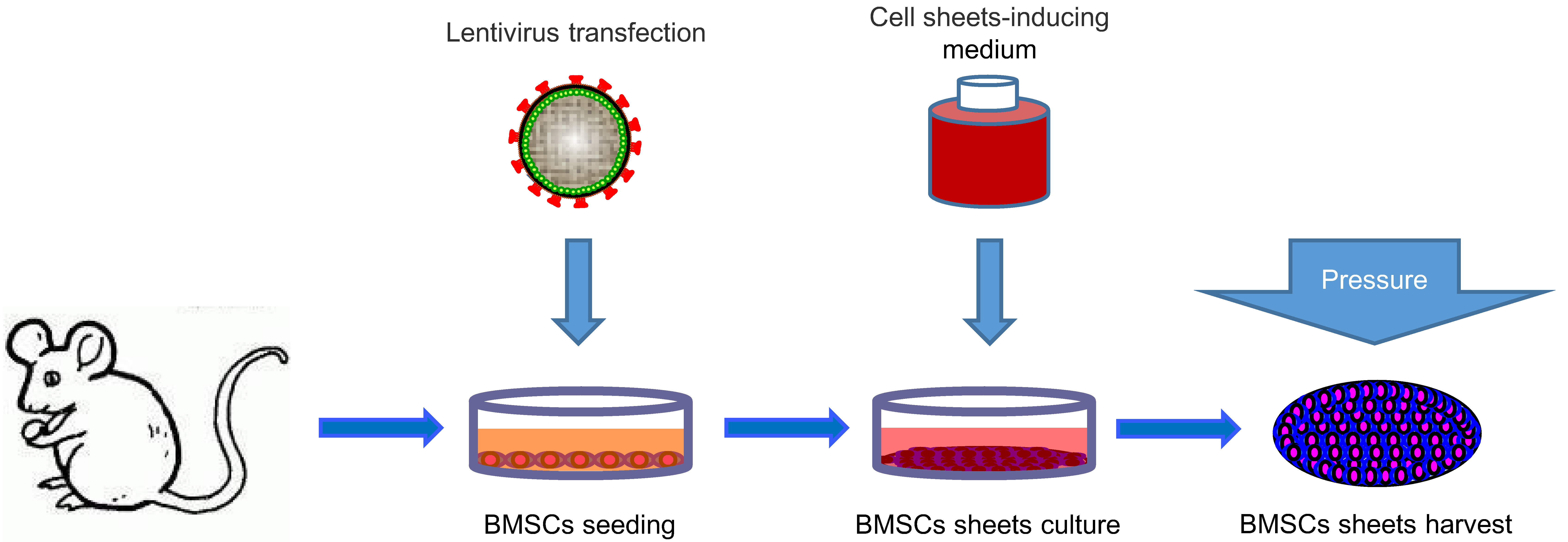


Figure S8. Schematic diagram of lentivirus transfected rat BMSCs.


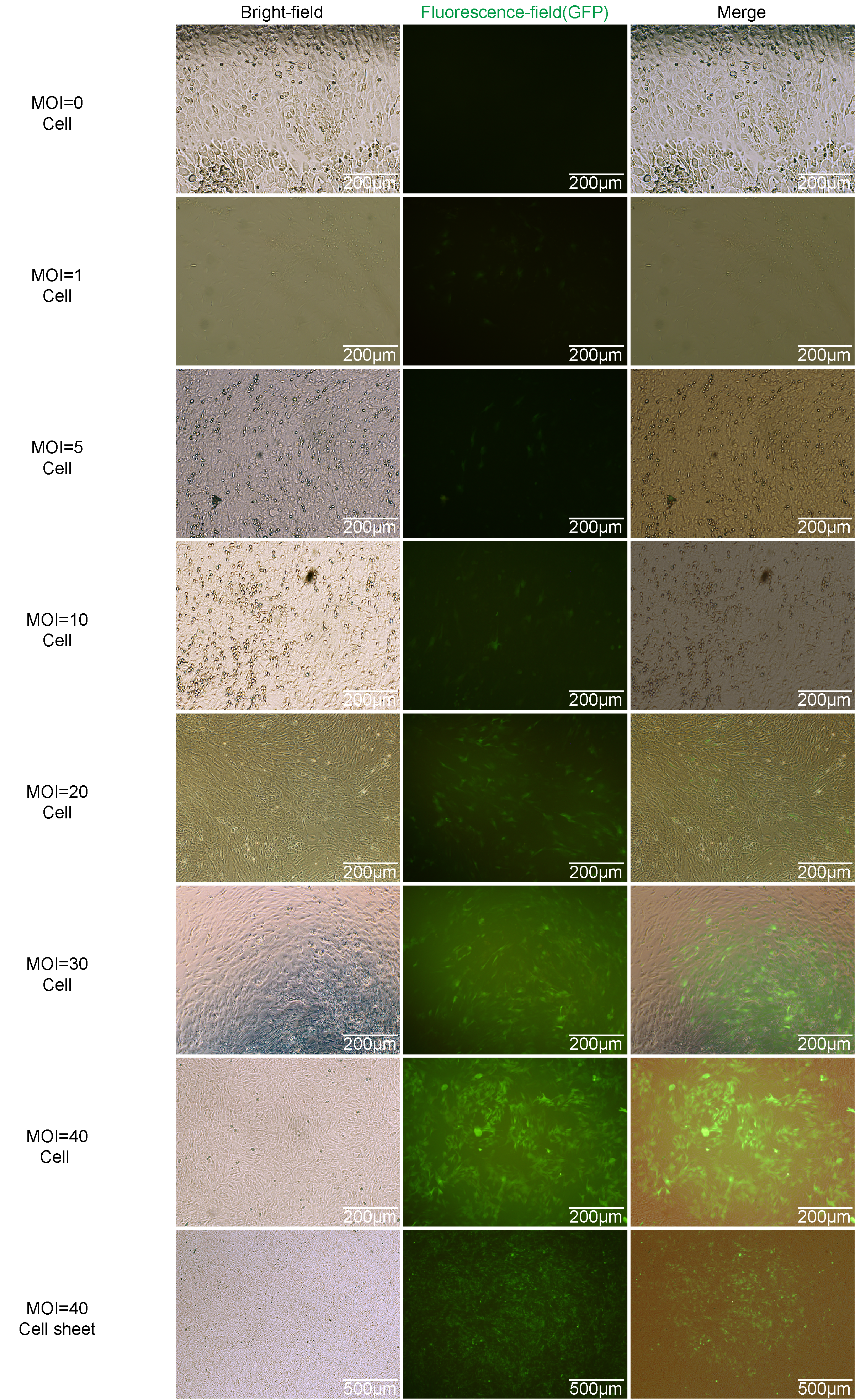


Figure S9. Lentiviral concentration screening for rat BMSCs sheet transfecting.


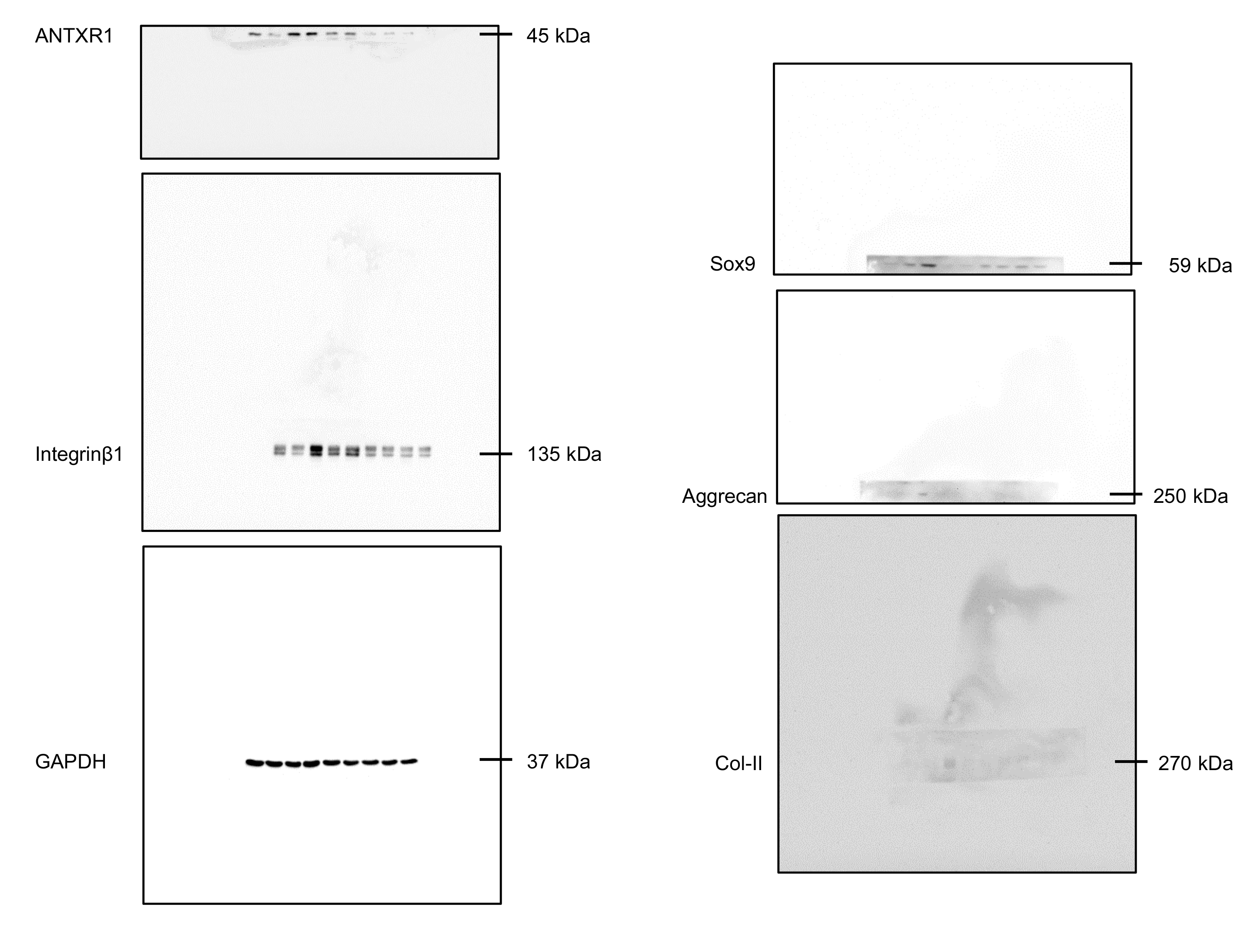


**Figure S10. Uncropped Western blots for Figure 2b.**


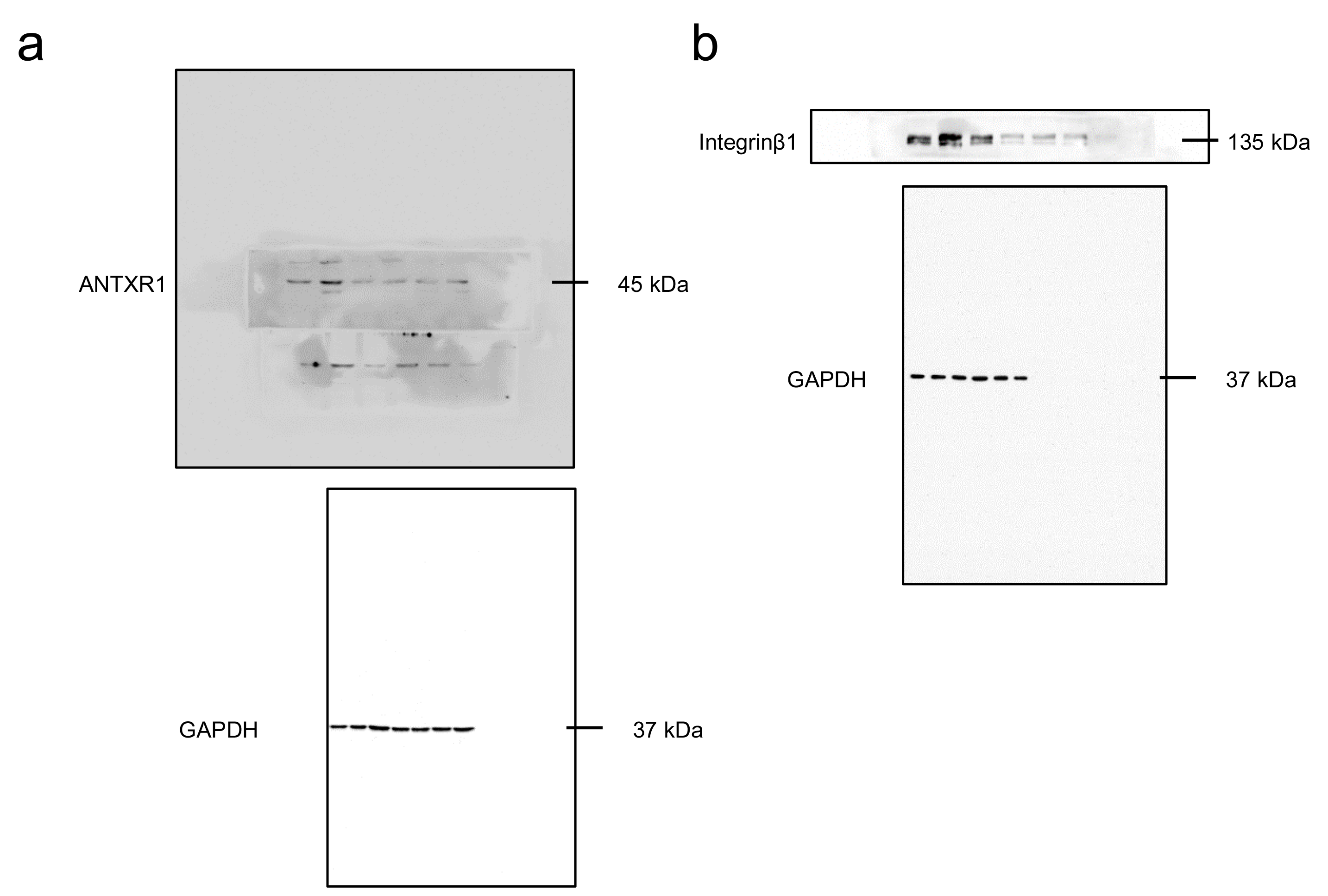


**Figure S11.** **Uncropped Western blots for Figure 3.** (a) Uncropped Western blots for Figure 3c. (b) Uncropped Western blots for Figure 3g.


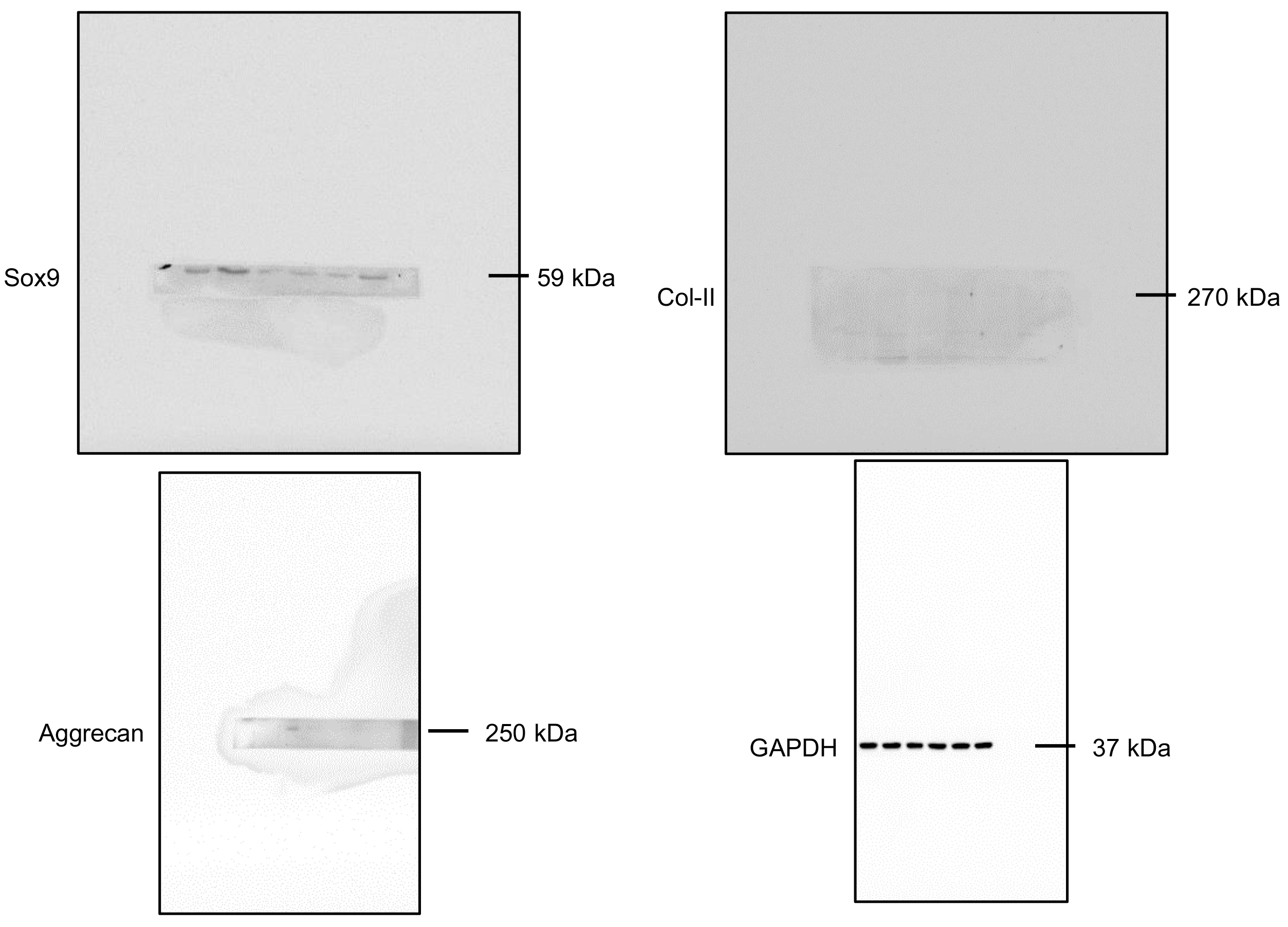


**Figure S12.** **Uncropped Western blots for Figure 4.**


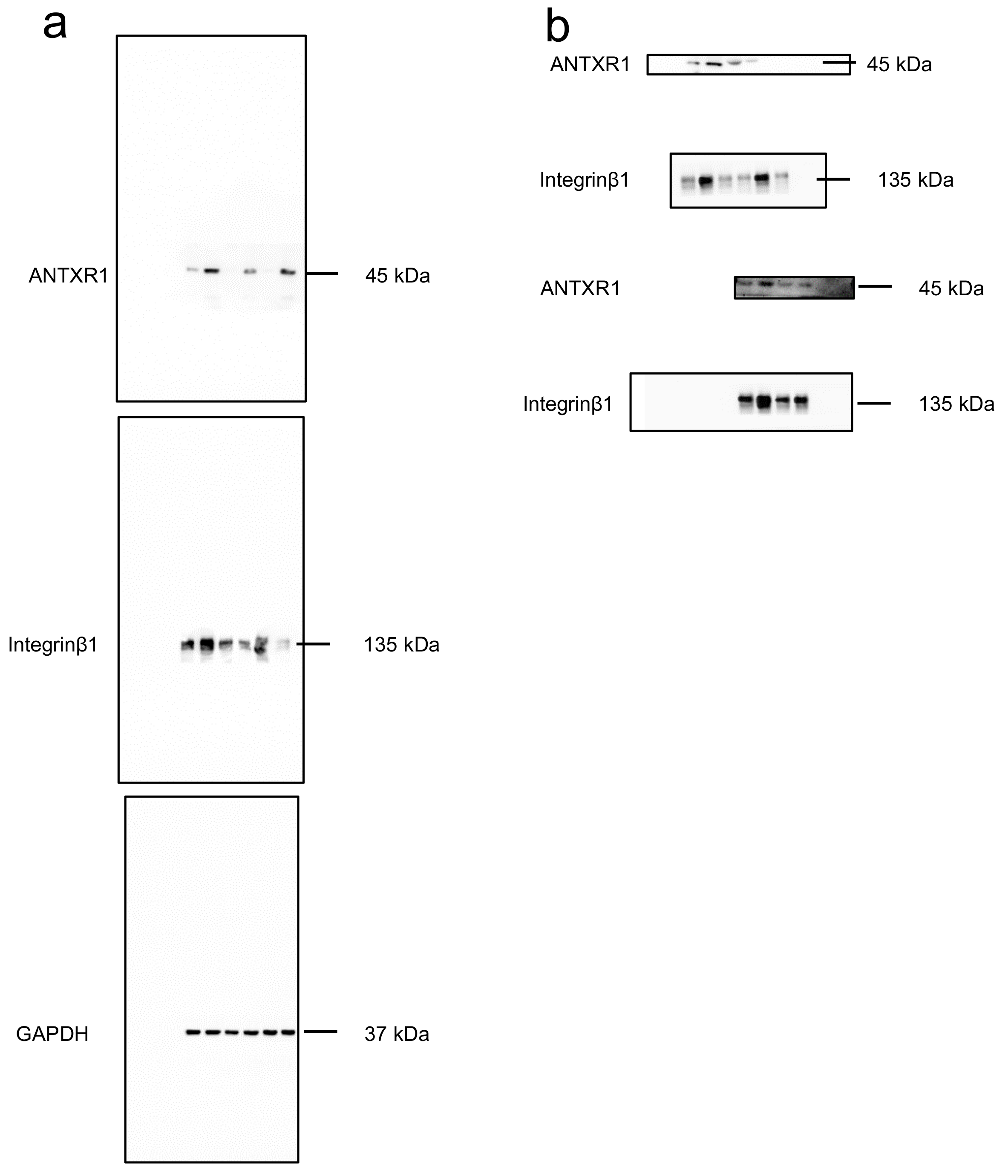


**Figure S13.** **Uncropped Western blots for Figure 5.** (a) Uncropped Western blots for Figure 5b. (b) Uncropped Western blots for Figure 5d.


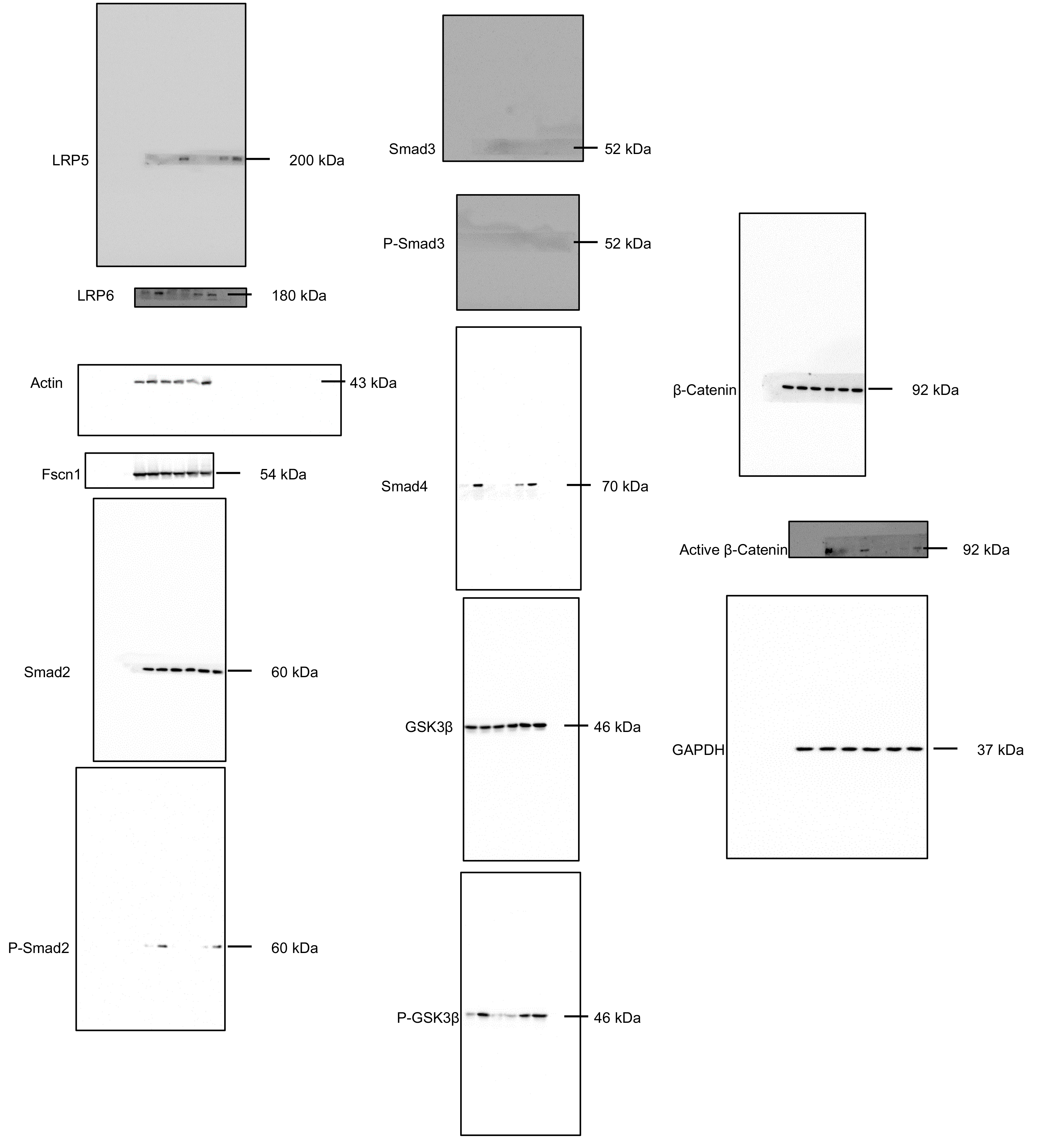


**Figure S14.** **Uncropped Western blots for Figure 7a**

**
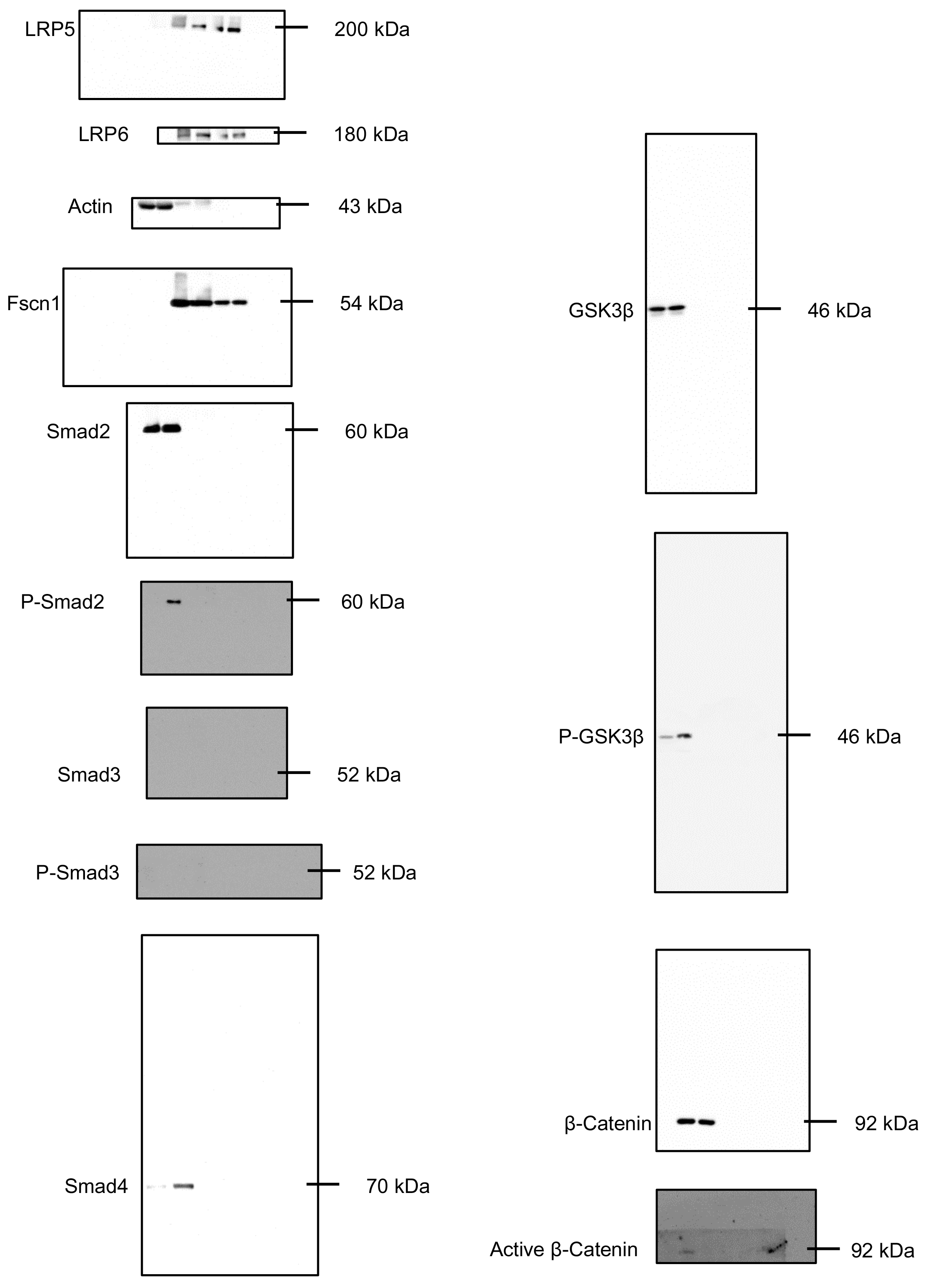
**

**Figure S15.** **Uncropped Western blots for Figure 7c.**

**Table S2. Proteins variation in the cell leading edge pathway analyzed by GO software.**

| **IPI_ID** | **Gene Name** | **Log2 ration of H/L** |
| --- | --- | --- |
| IPI00268688 | CD2-associated protein | -0.442972661 |
| IPI00556875 | RAB34, member of RAS oncogene family | -0.425809566 |
| IPI00929758 | talin 1 | 0.319878869 |
| IPI00753652 | actin related protein 2/3 complex, subunit 3 | 0.330691279 |
| IPI00115627 | ARP3 actin-related protein 3 homolog (yeast) | 0.345344664 |
| IPI00661414 | predicted gene 5492; actin related protein 2/3 complex, subunit 2 | 0.370087725 |
| IPI00130948 | SWA-70 protein | 0.400780671 |
| IPI00123181 | myosin, heavy polypeptide 9, non-muscle | 0.410317756 |
| IPI00458127 | drebrin-like | 0.430770507 |
| IPI00759948 | gelsolin | 0.433421534 |
| IPI00399943 | predicted gene 16372; actin related protein 2/3 complex, subunit 5 | 0.494978837 |
| IPI00830707 | cadherin 2; similar to N-cadherin | 0.513982328 |
| IPI00112963 | catenin (cadherin associated protein), alpha 1 | 0.59811692 |
| IPI00776140 | capping protein (actin filament) muscle Z-line, beta | 0.647024043 |
| IPI00653749 | abl-interactor 1 | 0.72113344 |
| IPI00676489 | catenin (cadherin associated protein), delta 1 | 0.731855175 |
| IPI00857569 | dynactin 1 | 0.84305855 |
| IPI00230205 | podoplanin | 0.864598909 |
| IPI00309262 | cyclin-dependent kinase 5 | 0.963408558 |
| IPI00125899 | catenin (cadherin associated protein), beta 1 | 1.360964842 |
| IPI00229069 | anthrax toxin receptor 1 | 1.61503651 |

**Table S3. Proteins variation in the actin cytoskeleton pathway analyzed by GO software.**

| **IPI_ID** | **Gene Name** | **Log2 ration of H/L** |
| --- | --- | --- |
| IPI00111265 | capping protein (actin filament) muscle Z-line, alpha 2 | 0.391179759 |
| IPI00112963 | catenin (cadherin associated protein), alpha 1 | 0.59811692 |
| IPI00115528 | plastin 3 (T-isoform) | 0.443757551 |
| IPI00115627 | ARP3 actin-related protein 3 homolog (yeast) | 0.345344664 |
| IPI00118895 | similar to beta-2-syntrophin; syntrophin, basic 2 | 0.412649733 |
| IPI00118899 | actinin alpha 4 | -0.897125709 |
| IPI00119111 | similar to calponin 3, acidic; predicted gene 4815; calponin 3, acidic | -0.342324285 |
| IPI00123181 | myosin, heavy polypeptide 9, non-muscle | 0.410317756 |
| IPI00123319, IPI00874728 | tropomyosin 2, beta | 0.438608201 |
| IPI00124819 | coronin, actin binding protein 1B | 0.49879819 |
| IPI00124820 | coronin, actin binding protein 1C; predicted gene 5790 | -0.580893557 |
| IPI00125091 | LIM and SH3 protein 1 | 0.340202468 |
| IPI00125880 | protein kinase C and casein kinase substrate in neurons 2 | -0.608544305 |
| IPI00135655 | vesicle-associated membrane protein, associated protein B and C | 0.362612363 |
| IPI00137087 | ADP-ribosylation factor guanine nucleotide-exchange factor 2 (brefeldin A-inhibited) | 0.777147645 |
| IPI00137331 | CAP, adenylate cyclase-associated protein 1 (yeast) | 0.444840634 |
| IPI00177038 | ARP2 actin-related protein 2 homolog (yeast); predicted gene 6828 | 0.350031208 |
| IPI00177235 | hypothetical protein LOC100044217; autophagy-related 4C (yeast) | 0.898560967 |
| IPI00229069 | anthrax toxin receptor 1 | 1.61503651 |
| IPI00229295 | erythrocyte protein band 4.1-like 3 | 0.519633924 |
| IPI00229534 | myristoylated alanine rich protein kinase C substrate | 0.508987512 |
| IPI00230044 | predicted gene 7848; predicted gene 7839; predicted gene 4157; similar to tropomyosin 3, gamma; tropomyosin 3, gamma; predicted gene 4903 | 0.831923297 |
| IPI00230707 | tyrosine 3-monooxygenase/tryptophan 5-monooxygenase activation protein, gamma polypeptide | 0.325202549 |
| IPI00263048 | nuclear mitotic apparatus protein 1 | 1.240758936 |
| IPI00277930 | capping protein (actin filament), gelsolin-like | 0.468459047 |
| IPI00311344 | caldesmon 1 | -0.451090444 |
| IPI00312067 | inositol polyphosphate phosphatase-like 1 | 0.301924014 |
| IPI00314844 | twinfilin, actin-binding protein, homolog 1 (Drosophila); predicted gene 4887 | -0.366101043 |
| IPI00317134 | cytoskeleton associated protein 5 | 0.52272054 |
| IPI00330862 | ezrin; hypothetical protein LOC100044177 | 0.291955409 |
| IPI00353420 | utrophin | 0.372079704 |
| IPI00380436 | actinin, alpha 1 | -0.497440543 |
| IPI00399943 | predicted gene 16372; actin related protein 2/3 complex, subunit 5 | 0.494978837 |
| IPI00408207 | myosin ID | 1.169816892 |
| IPI00458127 | drebrin-like | 0.430770507 |
| IPI00460717 | dystonin; hypothetical protein LOC100047109 | 0.906551627 |
| IPI00467104 | flightless I homolog (Drosophila); similar to cytoskeletal actin-modulating protein | -0.551184424 |
| IPI00467172 | similar to nuclear myosin I beta; myosin IC | 0.670906794 |
| IPI00515398 | myosin, heavy polypeptide 10, non-muscle | 0.303773025 |
| IPI00661414 | predicted gene 5492; actin related protein 2/3 complex, subunit 2 | 0.370087725 |
| IPI00751146 | twinfilin, actin-binding protein, homolog 2 (Drosophila) | 0.410439756 |
| IPI00753652 | actin related protein 2/3 complex, subunit 3 | 0.330691279 |
| IPI00753917 | filamin C, gamma | 0.473965367 |
| IPI00759948 | gelsolin | 0.433421534 |
| IPI00761270 | CAP-GLY domain containing linker protein 1 | 0.824237229 |
| IPI00776140 | capping protein (actin filament) muscle Z-line, beta | 0.647024043 |
| IPI00788359 | microtubule-associated protein 6 | -0.692122917 |
| IPI00828338 | four and a half LIM domains 3 | 0.309824587 |
| IPI00856926 | palladin, cytoskeletal associated protein | 0.470394191 |
| IPI00874516 | LIM domain and actin binding 1 | -2.756388272 |
| IPI00877182 | capping protein (actin filament) muscle Z-line, alpha 1; similar to capping protein (actin filament) muscle Z-line, alpha 1; predicted gene 3608; predicted gene 5920 | 0.636655687 |
| IPI00882056 | microtubule-associated protein, RP/EB family, member 2 | 0.303774922 |
| IPI00929758 | talin 1 | 0.319878869 |

**Table S4. Proteins variation in the actin binding pathway analyzed by GO software.**

| **IPI_ID** | **Gene Name** | **Log2 ration of H/L** |
| --- | --- | --- |
| IPI00111265 | capping protein (actin filament) muscle Z-line, alpha 2 | 0.391179759 |
| IPI00112963 | catenin (cadherin associated protein), alpha 1 | 0.59811692 |
| IPI00115528 | plastin 3 (T-isoform) | 0.443757551 |
| IPI00115627 | ARP3 actin-related protein 3 homolog (yeast) | 0.345344664 |
| IPI00118895 | similar to beta-2-syntrophin; syntrophin, basic 2 | 0.412649733 |
| IPI00118899 | actinin alpha 4 | -0.897125709 |
| IPI00119111 | similar to calponin 3, acidic; predicted gene 4815; calponin 3, acidic | -0.342324285 |
| IPI00123181 | myosin, heavy polypeptide 9, non-muscle | 0.410317756 |
| IPI00123319 | tropomyosin 2, beta | 1.355296213 |
| IPI00124819 | coronin, actin binding protein 1B | 0.49879819 |
| IPI00124820 | coronin, actin binding protein 1C; predicted gene 5790 | -0.580893557 |
| IPI00125091 | LIM and SH3 protein 1 | 0.340202468 |
| IPI00137331 | CAP, adenylate cyclase-associated protein 1 (yeast) | 0.444840634 |
| IPI00177038 | ARP2 actin-related protein 2 homolog (yeast); predicted gene 6828 | 0.350031208 |
| IPI00229069 | anthrax toxin receptor 1 | 1.61503651 |
| IPI00229295 | erythrocyte protein band 4.1-like 3 | 0.519633924 |
| IPI00229534 | myristoylated alanine rich protein kinase C substrate | 0.508987512 |
| IPI00230044 | predicted gene 7848; predicted gene 7839; predicted gene 4157; similar to tropomyosin 3, gamma; tropomyosin 3, gamma; predicted gene 4903 | 0.831923297 |
| IPI00230707 | tyrosine 3-monooxygenase/tryptophan 5-monooxygenase activation protein, gamma polypeptide | 0.325202549 |
| IPI00277930 | capping protein (actin filament), gelsolin-like | 0.468459047 |
| IPI00311344 | caldesmon 1 | -0.451090444 |
| IPI00312067 | inositol polyphosphate phosphatase-like 1 | 0.301924014 |
| IPI00314844 | twinfilin, actin-binding protein, homolog 1 (Drosophila); predicted gene 4887 | -0.366101043 |
| IPI00330862 | ezrin; hypothetical protein LOC100044177 | 0.291955409 |
| IPI00353420 | utrophin | 0.372079704 |
| IPI00380436 | actinin, alpha 1 | -0.497440543 |
| IPI00399943 | predicted gene 16372; actin related protein 2/3 complex, subunit 5 | 0.494978837 |
| IPI00408207 | myosin ID | 1.169816892 |
| IPI00458127 | drebrin-like | 0.430770507 |
| IPI00460717 | dystonin; hypothetical protein LOC100047109 | 0.906551627 |
| IPI00467104 | flightless I homolog (Drosophila); similar to cytoskeletal actin-modulating protein | -0.551184424 |
| IPI00467172 | similar to nuclear myosin I beta; myosin IC | 0.670906794 |
| IPI00515398 | myosin, heavy polypeptide 10, non-muscle | 0.303773025 |
| IPI00661414 | predicted gene 5492; actin related protein 2/3 complex, subunit 2 | 0.370087725 |
| IPI00751146 | twinfilin, actin-binding protein, homolog 2 (Drosophila) | 0.410439756 |
| IPI00753652 | actin related protein 2/3 complex, subunit 3 | 0.330691279 |
| IPI00753917 | filamin C, gamma | 0.473965367 |
| IPI00759948 | gelsolin | 0.433421534 |
| IPI00776140 | capping protein (actin filament) muscle Z-line, beta | 0.647024043 |
| IPI00828338 | four and a half LIM domains 3 | 0.309824587 |
| IPI00856926 | palladin, cytoskeletal associated protein | 0.470394191 |
| IPI00874516 | LIM domain and actin binding 1 | -2.756388272 |
| IPI00874728 | tropomyosin 2, beta | 1.248225741 |
| IPI00877182 | capping protein (actin filament) muscle Z-line, alpha 1; similar to capping protein (actin filament) muscle Z-line, alpha 1; predicted gene 3608; predicted gene 5920 | 0.636655687 |
| IPI00929758 | talin 1 | 0.319878869 |

**Table S5. Sequences of primers used in this study.**

| **Experiment** | **Description** | **Sequence (5’-3’)** | |
| --- | --- | --- | --- |
| Real-time PCR | ANTXR1 | F：CGCCTCTTACTACGGTGGAC | R：TAGCTTCGCCCCTTCTTCTG |
| Real-time PCR | Integrinβ1 | F：AAGTGAACAGTGAAGACAT | R：CTATCGCAGTTGAAGTTATC |
| Real-time PCR | Sox9 | F：AAAGACCACCCCGATTACAAG | R：TGAAGATGGCGTTAGGAGAGA |
| Real-time PCR | Aggrecan | F：CTGAAGTGGGGACCACAGTC | R：CTGAAGTGGGGACCACAGTC |
| Real-time PCR | Col-II | F：CGCTCAAGTCGCTGAACAACC | R：CAATCCAGTAGTCTCCGCTCTTCC |
| Real-time PCR | GAPDH | F：CAGCCGCATCTTCTTGTGC | R：CAGCCGCATCTTCTTGTGC |
| Knockdown | Integrinβ1-ShRNA1 | AACCACAGAAGTTTACATTAA | |
| Knockdown | Integrinβ1-ShRNA2 | TACTGCAAGAATGGAGTGAAT | |
| Knockdown | Integrinβ1-ShRNA3 | AATGCCAAATCTTGTGGAGAA | |
| Knockdown | ANTXR1-ShRNA1 | GGGACAACTTTAATGAAACTA | |
| Knockdown | ANTXR1-ShRNA2 | GGATTTCAATGAAACTCAGTT | |
| Knockdown | ANTXR1-ShRNA3 | CCTTTGCTGTAGAAGATACTT | |
| Knockdown | ANTXR1-ShRNA4 | CCCAACCAAGAACAATTTGAA | |
| Knockdown | Scramble-ShRNA | TTCTCCGAACGTGTCACGT | |

**Table S6. Detailed information on the antibodies used in this study**

| **Name** | **Supplier** | **Catalog number** | **WB dilution** | **IF dilution** | **IP dosage** | **Flow cytometry dilution** |
| --- | --- | --- | --- | --- | --- | --- |
| GAPDH | Cowin Biotech | CW0100M | 1/4000 |  |  |  |
| ANTXR1 | Abcam | ab21270 | 1/1000 | 1/50 | 10 µL |  |
| Integrinβ1 | Santa Cruz | sc-53711 | 1/100 | 1/10 | 10 µL |  |
| Sox9 | Novus | NBP1-85551 | 1/200 |  |  |  |
| Aggrecan | Novus | NB600-504 | 1/100 |  |  |  |
| Col-II | Novus | NB600-844 | 1/200 |  |  |  |
| LRP5 | Cell Signaling | 5731 | 1/1000 |  |  |  |
| LRP6 | Abcam | ab134146 | 1/4000 |  |  |  |
| Actin | Abcam | ab205 | 1/200 |  |  |  |
| Fscn1 | Abcam | ab126772 | 1/20000 |  |  |  |
| Smad2 | Cell Signaling | 5339 | 1/1000 |  |  |  |
| P-Smad2 | Cell Signaling | 3108 | 1/1000 |  |  |  |
| Smad3 | Cell Signaling | 9523 | 1/1000 |  |  |  |
| P-Smad3 | Cell Signaling | 9520 | 1/1000 |  |  |  |
| Smad4 | Cell Signaling | 38454 | 1/1000 |  |  |  |
| GSK3β | Cell Signaling | 9832 | 1/1000 |  |  |  |
| P-GSK3β | Cell Signaling | 11757 | 1/1000 |  |  |  |
| β-Catenin | Cell Signaling | 8480 | 1/1000 |  |  |  |
| Active β-Catenin | Sigma-Aldrich | 05665 | 1/1000 |  |  |  |
| Mouse mAb IgG Isotype Control | Cell Signaling | 5415 |  |  | 10 µL |  |
| Cy™3 AffiniPure Goat Anti-Rabbit IgG (H+L) | Jackson | 111-165-003 |  | 1/200 |  |  |
| Goat Anti-Mouse IgG, Cy3 Conjugated | Jackson | 115-165-003 |  | 1/200 |  |  |
| Goat Ant-Rabbit IgG, HRP Conjuagted | Zhuangzhi Bio | EK020 | 1/40000 |  |  |  |
| Goat Ant-mouse IgG, HRP Conjuagted | Zhuangzhi Bio | EK010 | 1/40000 |  |  |  |
| Anti-Rat CD44 | BD Biosciences | 550974 |  |  |  | 1/100 |
| Anti-Rat CD45 | BD Biosciences | 554878 |  |  |  | 1/100 |
| Anti-Rat CD90 | BD Biosciences | 554898 |  |  |  | 1/100 |

Table S7. Values of coefficients calculated on images with ANTXR1-Integrinβ1 localization show in Fig. 6a

| Coefficient | ANTXR1-Integrinβ1  (Control) | ANTXR1-Integrinβ1  (120KPa, 1h) |
| --- | --- | --- |
| Pearson's correlation coefficient (Rr) | 0.51±0.05 | 0.52±0.04 |
| Manders’ overlap coefficient (R) | 0.64±0.03 | 0.71±0.05 |
| Overlap coefficients k1 and k2 | k1=0.68±0.10  k2=0.74±0.02 | k1=0.63±0.05  k2=0.60±0.06 |
| Co-localization coefficients m1 and m2 | m1=0.92±0.04  m2=0.57±0.06 | m1=0.93±0.05  m2=0.79±0.04 |
| Co-localization coefficients M1 and M2 | M1=0.70±0.05  M2=0.51±0.08 | M1=0.90±0.05  M2=0.67±0.06 |

*Note: three images from three sections were quantified. Background was corrected in manual mode using selected ROI.*

Table S8. Values of coefficients calculated on images with ANTXR1-Phalloidin localization show in Fig. 6b

| Coefficient | ANTXR1-Phalloidin  (Control) | ANTXR1-Phalloidin  (120KPa, 1h) |
| --- | --- | --- |
| Pearson's correlation coefficient (Rr) | 0.69±0.04 | 0.74±0.05 |
| Manders’ overlap coefficient (R) | 0.71±0.06 | 0.78±0.04 |
| Overlap coefficients k1 and k2 | k1=0.61±0.03  k2=0.70±0.09 | k1=0.91±0.05  k2=0.66±0.04 |
| Co-localization coefficients m1 and m2 | m1=0.95±0.05  m2=0.91±0.04 | m1=0.98±0.04  m2=0.87±0.04 |
| Co-localization coefficients M1 and M2 | M1=0.76±0.04  M2=0.80±0.06 | M1=0.71±0.05  M2=0.63±0.08 |

*Note: three images from three sections were quantified. Background was corrected in manual mode using selected ROI.*

**SUPPLEMENTARY REFERENCES**

1. Huang, L. *et al.* The effects of static pressure on chondrogenic and osteogenic differentiation in condylar chondrocytes from temporomandibular joint. *Arch Oral Biol* **60**, 622-630**,** doi:10.1016/j.archoralbio.2015.01.003 (2015).

2. Zhang, M. *et al.* Effects of condylar elastic properties to temporomandibular joint stress. *J Biomed Biotechnol* **2009**, 509848**,** doi:10.1155/2009/509848 (2009).

3. Kuroda, S. *et al.* Biomechanical and biochemical characteristics of the mandibular condylar cartilage. *Osteoarthritis Cartilage* **17**, 1408-1415**,** doi:10.1016/j.joca.2009.04.025 (2009).

4. Mow, V. C., Ateshian, G. A. & Spilker, R. L. Biomechanics of diarthrodial joints: a review of twenty years of progress. *J Biomech Eng* **115**, 460-467 (1993).

5. Zhao, Y. H. *et al.* Hydrostatic pressure promotes the proliferation and osteogenic/chondrogenic differentiation of mesenchymal stem cells: The roles of RhoA and Rac1. *Stem Cell Res* **14**, 283-296**,** doi:10.1016/j.scr.2015.02.006 (2015).
